# Supplementary material for: Task state representations in vmPFC mediate relevant and irrelevant value signals and their behavioral influence
Source: Nat Commun. 2023 May 31;14:3156. doi: 10.1038/s41467-023-38709-w (PMC10232498; doi:10.1038/s41467-023-38709-w)
Supplement: Supplementary file 1 — Supplementary Information [file 41467_2023_38709_MOESM1_ESM.pdf]

Supplementary Information

- Fig. S1: Full procedure and experimental design for all phases, related to Fig 1
- Fig. S2: Nested RT models (EV, Context, Block and switch) related to Fig 2
- Fig. S3: Alternative RT models, extended RT model comparisons and correlation matrix of all regressors, related to Fig 2
- Fig. S4: Exploratory analysis of RT model presented in Main Text, related to Fig 2
- Fig. S5: Behavioral accuracy results: related to Fig 2
- Fig. S6: Frequency bias in the design and supplementary information for Representational Similarity Analysis: related to Fig. 5 and Fig. 3
- Fig. S7: Supplementary information for Value similarity analysis: related to Fig. 4 and Fig. 5
- Fig. S8:Supplementary information for perceptual similarity analysis: related to Fig. 4 and Fig. 5
- Fig. S9: Modelling probability assigned to the EV class: related to Fig. 5
- Fig. S10: Main effects and corresponding data, fMRI effects, related to Fig. 5
- Fig. S11: Main effects and corresponding data, link of fMRI to behavioral accuracy, related to Fig. 6
- Fig. S12: Main univariate results
- Fig. S13: Additional univariate results,
- Table S1: Detailed univariate results: Clusters for whole brain univariate analysis
- Effect sizes and confidence intervals for best explaining models:
  - Table S2: RT model
  - Table S3: fMRI model (P_{EV}, main model)
  - Table S4: fMRI model (P_{EV}, nested in EV_{back})
  - Table S5: RSA model - Main effect models
  - Table S6: RSA model - Main effect models value difference models

Source data for all figures are provided as a Source Data file.

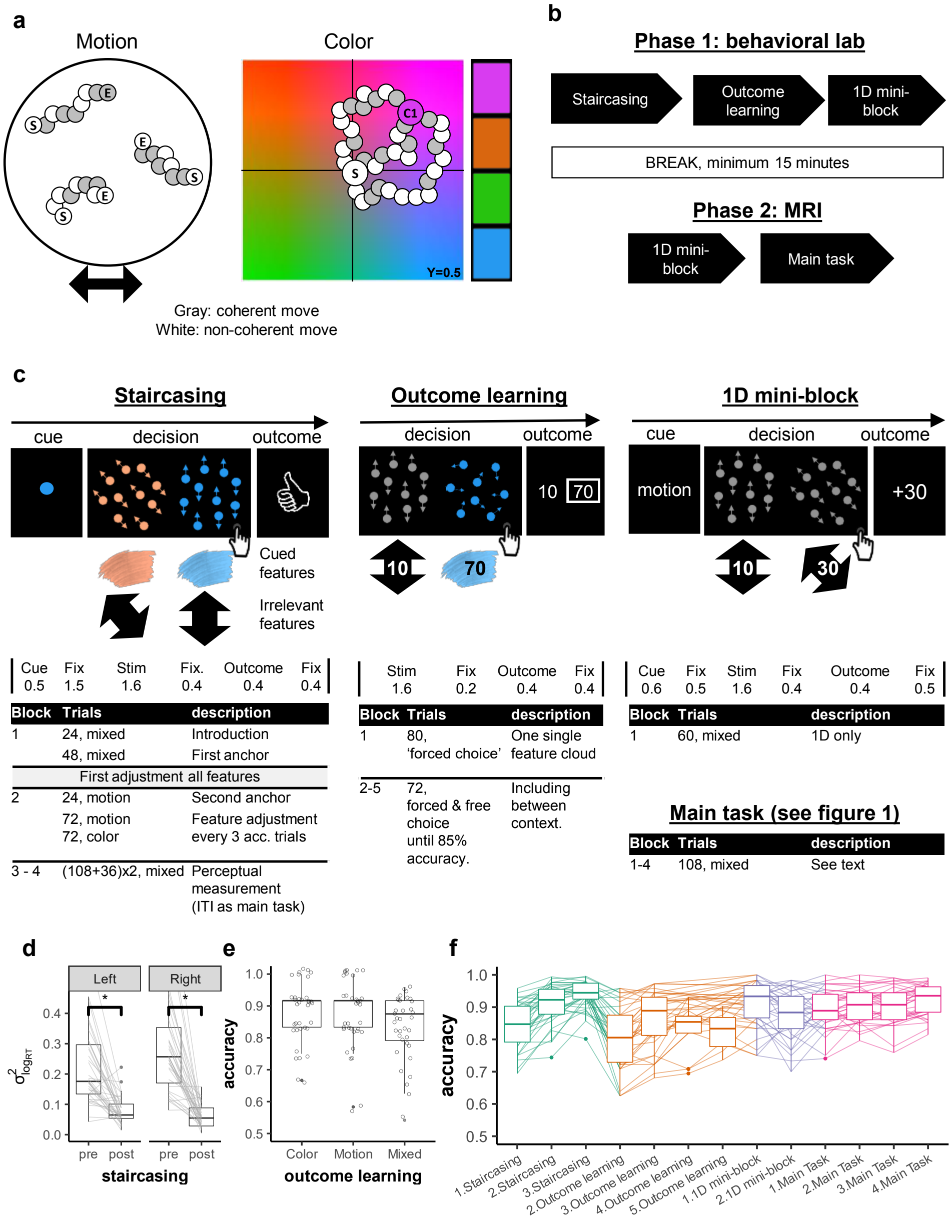

Figure S1: Full procedure and experimental design for all phases

Figure S1: Full procedure and experimental design for all phases, related to Fig 1. a. Brownian algorithm for color and motion. Each illustration shows the course of 3 example dots; 'S' and 'E' marked dots reflect Start and End positions, respectively. Remaining dots represent location in space for different frames. Left panel: Horizontal motion trial. Shown are framewise dot positions between start and end. In each frame, a different set of dots moved coherently in the designated direction (gray) with a fixed speed; remaining dots moved in a random direction [conceptually taken from [45]]. Right panel: Example of a pink color trial. We simulated the YCbCr color space that is believed to represent the human perception in a relative accurate way [cf. [65]]. A fixed luminance of Y = 0.5 was used. For technical reasons we sliced the X-axis by 0.1 on each side and the Y-axis by 0.2 from the bottom of the space to ensure the

middle of the space remained gray given the chosen luminance. In each frame, a different set of dots (always 30% of the dots) moved coherently towards the target color in a certain speed whereas the rest were assigned with a random direction. All target colors were offset by 23.75% from the center towards each corner. Right bar illustrates the used target colors. **b.** Full procedure. The experiment consisted of two phases, the first one took place in the behavioral lab and included Staircasig, Outcome-learning and the first 1D mini-block. The second took place inside the MRI scanner and consisted of the second 1D mini-block and the main task. **c.** Example trial procedures and timing of the different tasks. Timing of each trial is depicted below illustrations. **Staircasing (left)** Each trial started with a cue of the relevant feature. Each cloud had one or two features (motion and/or color) and participants had to detect the cued feature. Participants' task was to choose the cued feature (here: blue). After a choice, participants received feedback if they were correct and faster than 1 second, correct and slower, or wrong. **Outcome learning (middle)** Participants were presented with either one or two single-feature clouds and asked to chose the highest valued feature. Following their choice, they were presented with the values of both clouds, with the chosen cloud's associated value marked with a square around it. The pair of shown stimuli included across contexts comparisons, e.g. between up/right and blue, as shown. **1D mini block (right)** At the end of the first phase and beginning of the second phase participants completed a mini-block of 60 1D trials during the anatomical scan (30 color-only, 30 motion-only, interleaved). Participants were again asked to make a value-based two alternative forced choice choice decision. In each trial, they were first presented with a contextual cue (color/motion), followed by the presentation of two single-feature clouds of the cued context. After a choice, they were presented with the chosen-cloud's value. No BOLD response was measured during these blocks and timing of the trials was fixed and shorter than in the main task (see Main task preparation in methods) **Main task (bottom)** This part included 4 blocks, each consisting of 36 1D and 72 2D trials trials presented in an interleaved fashion (see method and Fig. 1). **d.** Button specific reduction in RT variance following the staircasing. We verified that the staircasing procedure also reduced differences in detection speed between features when testing each button separately. Depicted is the variance of reaction times (RTs) across different color and motion features (y axis). While participants' RTs were markedly different for different features before staircasing (pre), a significant reduction in RT differences was observed after the procedure (post, paired t-test: $p < .001$, N=35) **e.** Choice accuracy in outcome learning trials. Participants achieved near ceiling accuracy in choosing the highest valued feature in the outcome learning task, also when testing for color, motion and mixed trials separately (ps$< .001$, N=35). Mixed trials only appeared in this part of the experiment to encourage mapping of the values on similar scales. **f.** Accuracy throughout the experiment, plotted for each block of each part of the experiment. *In the staircasing (left)* High accuracy for the adjustment and measurement blocks (2-3) ensured that there were no difficulties in perceptual detection of the features. *In Outcome learning* a clear increase in accuracy throughout this task indicated learning of feature-outcome associations. Note that Block 5 of this part was only included for those who did not achieve 85% accuracy beforehand. Starting the *1D mini blocks* (middle) and throughout the*main task* (right) until the end of the experiment high accuracy. μ and σ from left to right: Staircasing:

.84,.07;.91,.06;.94,.04; Outcome Learning: .81,.1;.86,.09;.83,.08;.82,.06; 1D mini blocks: .91,.07;.88,.08; Main task: .89,.06;.91,.05;.9,.06;.92,.05.; N=35. In panels d-f boxes mid-line represent mean, lower and upper the 25th and 75th percentile and whiskers extend to the range of the data (no more than 1.5 of the full box range). Data beyond the whiskers are plotted individually as solid points. Source data are provided as a Source Data file.

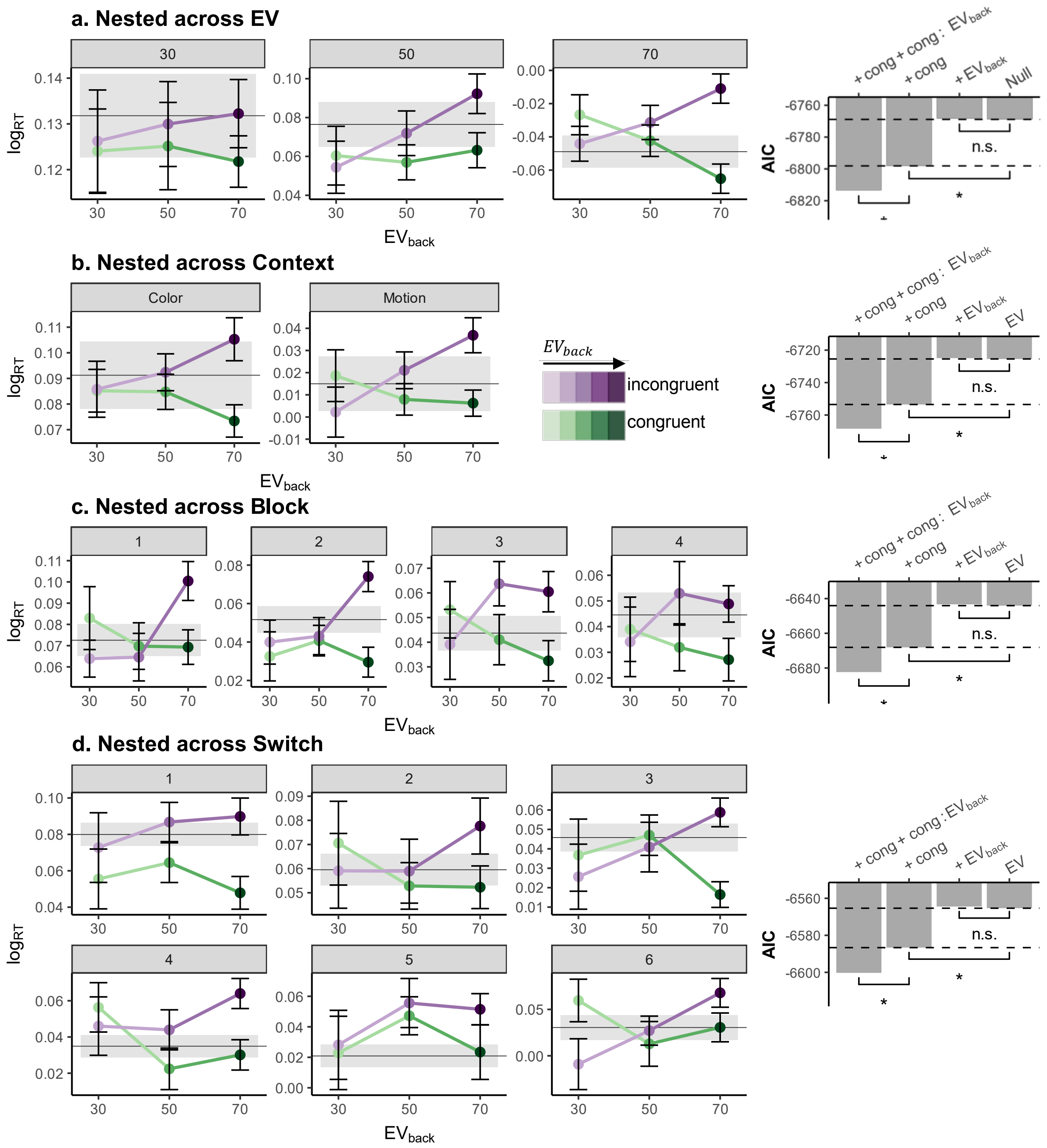

Figure S2: Nested RT models, related to Fig 2

Figure S2: Nested RT models, related to Fig 2

a-d. Nested models within Factors. Each row represents one congruency analysis, done separately for each level of expected value (a, top row), context (b, 2nd row), block (c, 3rd row) or switch (d, bottom row). The RT effect of Congruency × EV_{back} is shown on the left, corresponding AICs for mixed effect models with nested factors are shown on the right. Mean RT (line) and SEM (shades) for the

corresponding 1D trials is plotted in gray for each panel (e.g. mean across all 1D trials where EV=30 are on top left panel). Error bars assigned to colored lines and gray error band represent corrected within subject SEMs [46, 47]. Null models shown on the right are identical to Eq. 2, albeit included $\zeta_{0_{k_v}}$, which is the factor-specific (v) intercept nested within each within each subject level (see methods). Likelihood ratio tests were performed to asses improved model fit when adding (1) Congruency or (2) EV_{back} terms to the Null model and when adding (3) Congruency $\times$ EV_{back}) in addition to Congruency. Stars represent p values less than .05. For nested within EV, the Null model did not include a main effect for EV and the likelihood ratio (LR) tests with added term: (1) $\chi^2_{(1)} = 31.22$, $p < .001$; (2) $\chi^2_{(1)} = 1.47$, $p = .226$; (3) $\chi^2_{(1)} = 19.37$, $p < .001$; For models nested within Context the LR test was: (1) $\chi^2_{(1)} = 30.01$, $p < .001$; (2) $\chi^2_{(1)} = 1.5$, $p = .22$; (3) $\chi^2_{(1)} = 18.9$, $p < .001$; For models nested within Block: (1) $\chi^2_{(1)} = 26.06$, $p < .001$; (2) $\chi^2_{(1)} = 1.27$, $p = .26$; (3) $\chi^2_{(1)} = 18.25$, $p < .001$; And for models nested within switch: (1) $\chi^2_{(1)} = 23.29$, $p < .001$; (2) $\chi^2_{(1)} = 1.13$, $p = .29$; (3) $\chi^2_{(1)} = 17.66$, $p < .001$;, N=35 for all panels and models. In the first row (nested across EV) the interaction with EV is visible, i.e. the higher the EV, the stronger our effects of interests were. Source data are provided as a Source Data file.

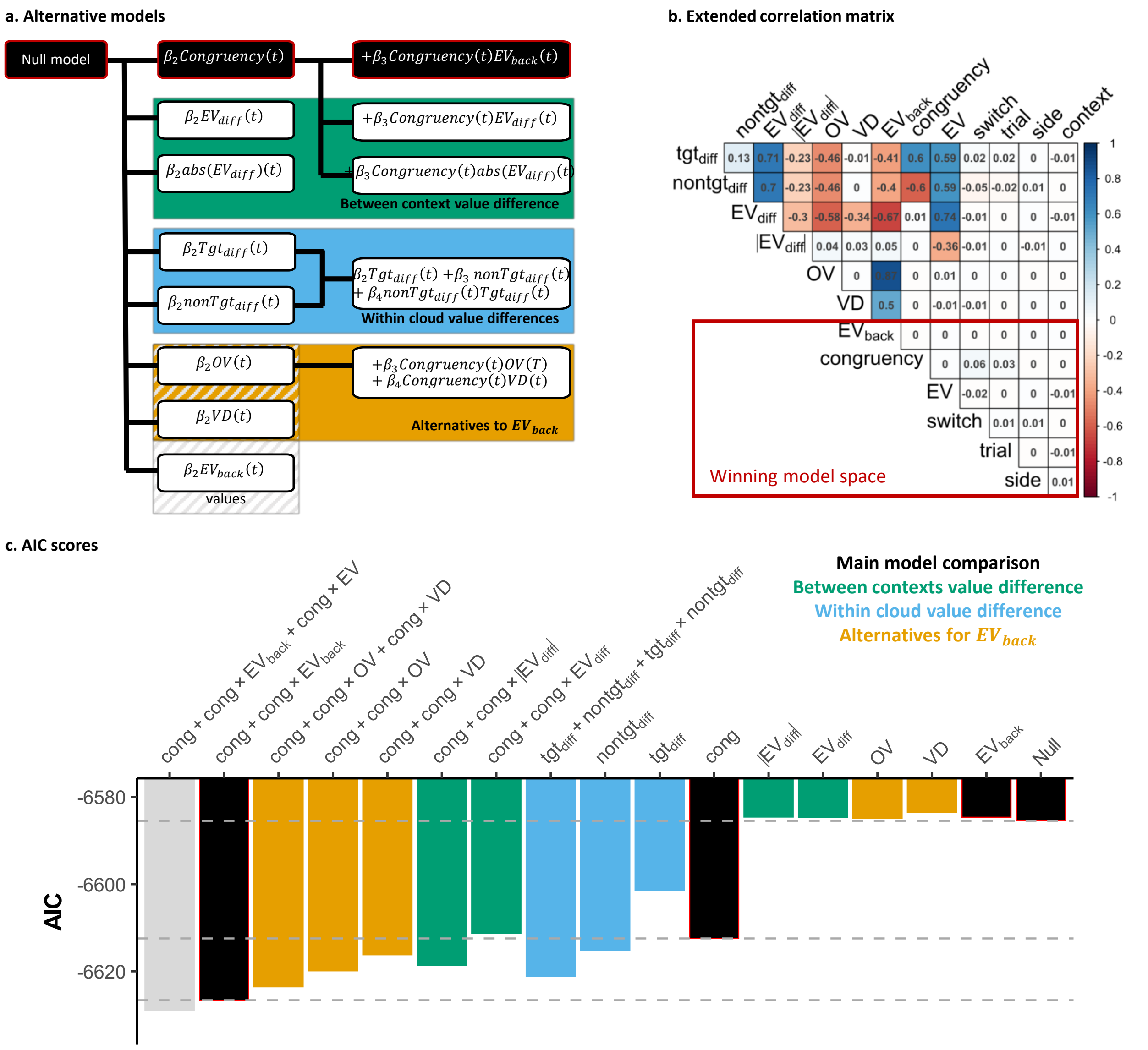

Figure S3: Alternative RT models, extended RT model comparisons and correlation matrix of all regressors, related to Fig 2.

Figure S3: Alternative RT models, extended RT model comparisons and correlation matrix of all regressors, related to Fig 2.
a. Alternative mixed effect models, each represented as a row which lists main factors of interest. We clustered different alternative models into three classes: *Green models* included factors that reflected the difference between the expected values of both contexts (EV - EV_{back}, including unsigned EV factors); *blue* models include instead factor that reflect the value-difference between context within each cloud where 'tgt' (target) is the chosen cloud with the highest value according to the relevant context and *orange* models included two alternative parameterization of values in the non-relevant context: irrelevant features' Value Difference (VD) and Overall Value (OV), which are also orthogonal to Congruency (Cong), and to each other. *In black* is the main model comparison as presented in the main text. **b. Extended**

correlation matrix. Averaged correlation across subjects of all scaled regressors for accurate 2D trials (models' input). Marked in red rectangle are main factors of the experiment which are orthogonal by design and used for the model comparison reported in the Main Text. **c. AIC scores.** We tested different alternatives shown in (a) in a stepwise hierarchical model comparison, as in the main text. Each bar represents the AIC (y-axis) of a different model (x-axis) where the labels on the x-axis depict the added terms to the Null model for that specific model. The Null model included nuisance regressors and the main effect of EV (see ν and β_1 in Eq. 2). The models described in the main text are shown in black. The gray model includes the additional term for Congruency $\times$ EV. Dashed lines correspond to the AIC values of the models used in the main text. Importantly, no main effect representing only the contextually irrelevant values (VD, OV, EV_{back}) nor the difference between the EVs (EV_{diff},$|\text{EV}_{\text{diff}}|$, also when excluding EV from the null model, not presented) improved model fit over the Null model. This supports our finding that neither large irrelevant values, nor their similarity to the objective EV, influenced participants' behavior. Similar to EV_{back}, factors from the green and orange clusters are also orthogonal to Congruency, which allowed us to test their interaction. Factors from the blue cluster highly correlate with both Congruency (and EV_{back}) and therefore were tested separately. Non of the alternatives provided a better AIC score (y axis, lower is better). Source data are provided as a Source Data file.

a

effect	Df	AIC	likelihood ratio test	P value
Switch	1	-6490.88	15.19	<.001
Trial	1	-6499.01	7.04	.008
Context	1	-6304.79	201.27	<.001
EV	1	-6159.75	346.31	<.001
Congruency	1	-6496.76	9.29	.002
Congruency x EV	1	-6501.69	4.37	.037
Congruency x EV_{back}	1	-6488.35	17.70	<.001
Switch x Side	1	-6502.42	3.64	.0565
Switch x Context	1	-6503.25	2.80	.094
Switch x EV	1	-6500.23	5.83	.0158
Switch x Congruency	1	-6502.54	3.52	.068
Context x EV	1	-6499.16	31.07	<.001
Context x Trial	1	-6499.16	6.89	.009
Side x EV_{back}	1	-6501.28	4.77	.029

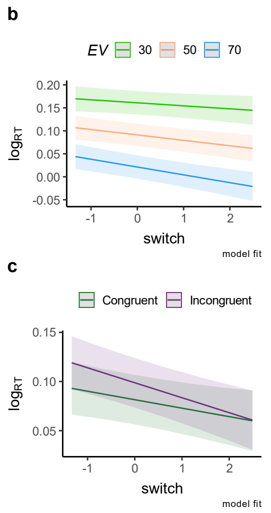

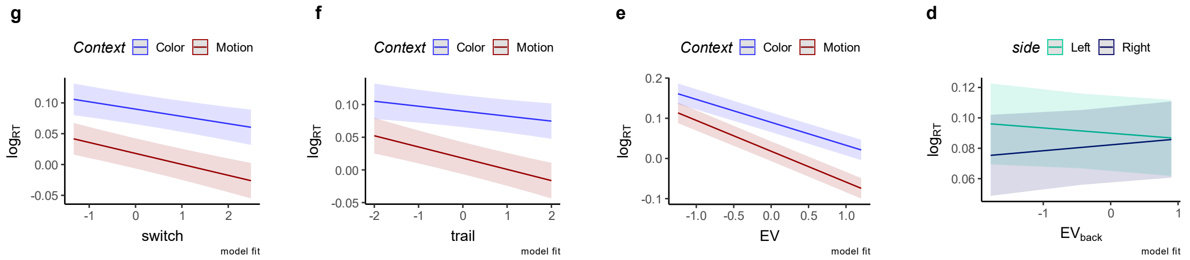

Figure S4: Exploratory analysis of RT model presented in Main Text, related to Fig 2.

Figure S4: Exploratory analysis of RT model presented in Main Text, related to Fig 2.
a. The table presents the individual contribution of terms taken from Eq. 2 and all possible two-way interactions to the model fit using the drop1 function in R [68]. In short, this exploratory analysis started with a model that included all main effects from Eq. 2 and all possible 2-way interaction between them and tested which terms contribute to the fit. If a term did not improve fit, it was dropped from the model. Presented are all effects with p value less than $p < .01$ for likelihood ratio test with added terms. Additionally, we specifically tested if the switch interacts with our main effect and found no such interaction (likelihood-ratio test with added term for Congruency x EV_{back} x switch:$\chi^2_{(1)} = 3.70$, $p = .157$). **b-g.** Model fits of all effects with $p < .01$ for likelihood ratio test with added terms. X-axes are normalized (as in the model) and y-axes reflect RTs on a log scale (model input). Clockwise from the top: RTs became progressively faster with increasing trials since the context switch. This effect was possibly stronger for higher EV (b) and for incongruent trials (c). We note that our experiment was not designed to test the effect of the switch. (d) An interaction of Side and EV_{back} was found, for which we offer no explanation. Panels (e) to (g) reflect interaction of context with EV (e), trial (f), and switch (g). In panels b-g error bands represent the 89% confidence interval. P values of each effect are found in the table in panel (a). We note that due to the used perceptual color space there might be a context-specific ceiling effect in RTs due to training throughout the task which could have induced effects of context. Specifically, since dots start gray and slowly 'gain' the color, it might take a few frames until there is

any evidence for color. However, the motion could be theoretically detected already on the second frame (since coherence was very high). This could explain why some effects that represent decrease in RT might hit a boundary for color (and not motion). Crucially, we refer the reader to supplementary Fig S2 where the main model comparison hold also when we ran the model nested within the levels of Context. Source data are provided as a Source Data file.

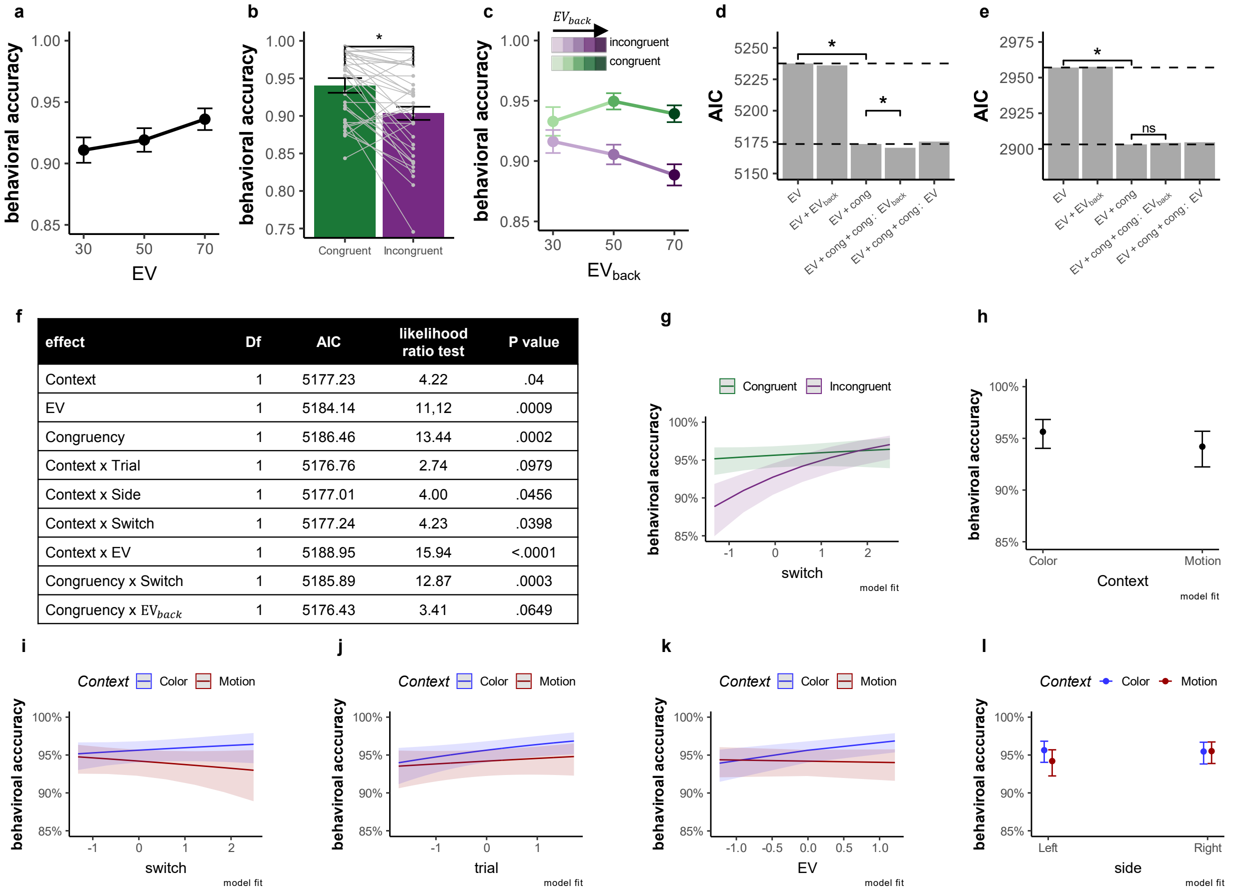

f

effect	Df	AIC	likelihood ratio test	P value
Context	1	5177.23	4.22	.04
EV	1	5184.14	11,12	.0009
Congruency	1	5186.46	13.44	.0002
Context x Trial	1	5176.76	2.74	.0979
Context x Side	1	5177.01	4.00	.0456
Context x Switch	1	5177.24	4.23	.0398
Context x EV	1	5188.95	15.94	<.0001
Congruency x Switch	1	5185.89	12.87	.0003
Congruency x EV_{back}	1	5176.43	3.41	.0649

Figure S5: Behavioral accuracy results: related to Fig 2.

Figure S5: Behavioral accuracy results: related to Fig 2.

a. Comparison of accuracy (y-axis) for each level of EV (x-axis) showed that participants were more accurate for higher EV, likelihood ratio test against null model: $p = .001$, N=35. **b.** Comparison of congruent versus incongruent trials also revealed a performance benefit of the former, paired t-test: $p = .001$, N=35. **c.** The effect of Congruency was modulated by EV_{back}, i.e. the more participants could expect to receive from the ignored context, the less accurate they were when the contexts disagreed (x axis, shades of colours). Further investigations revealed that the modulation of EV_{back} is likely limited to Incongruent trials (likelihood ratio test with added term: $\chi^2_{(1)} = 6.91$, $p = .009$, N=35, when modeling only Incongruent trials), yet does not increase accuracy for Congruent trials (likelihood ratio test with added term: $\chi^2_{(1)} = 0.07$, $p = .794$, N=35, when modeling only congruent trials), likely due to a ceiling effect. Error bars in panels a-c represent corrected within subject SEMs [46, 47]. **d.** Hierarchical model comparison of choice accuracy, similar to the RT model reported in the main text. These analyses showed that including Congruency improved model fit (likelihood-ratio test with added term: $p < .001$, N=35). Including the additional interaction of Congruency $\times$ EV_{back} improved the fit even more (likelihood-ratio test with added term: $p = .03$, N=35). **e.** We replicated the choice accuracy main effect in an independent sample of 21 participants outside of the MRI scanner, i.e. including Congruency improved model fit

(likelihood-ratio test with added term: $\chi^2_{(1)} = 55.95$, $p < .001$). We did not find a main effect of EV on accuracy in this sample (likelihood-ratio test with added term: $\chi^2_{(1)} = 0.93$, $p = .333$). The interaction term Congruency $\times$ EV_{back} did not significantly improve fit in this sample. Modeling only Incongruent trials, as above, reveled that EV_{back} had a marginal effect on accuracy (likelihood-ratio test with added term: $\chi^2_{(1)} = 2.90$, $p = .088$). Near-ceiling accuracies in Congruent trials in combination with a smaller sample might have masked the effects. **f.** The table presents the individual contribution of terms taken from Eq. 3 and all possible two-way interactions to the model fit using the drop1 function in R [68]. In short, this exploratory analysis started with a model that included all main effects from Eq. 3 and all possible 2-way interaction between them and tested which terms contribute to the fit. If a term did not improve fit, it was dropped from the model. Subsequent panels present all the effects corresponding to $p < .01$. Note that this is a non-hypothesis driven exploration of the data and that accuracy was very high in general throughout the main task. **g.** Accuracy as a function of time since switch. Akin to RTs, accuracy increased with number of trials since the last context switch, mainly for incongruent trials. **h.** Context effect on accuracy. According to the exploratory model, participants were slightly more accurate in color than in motion trials. However, a direct paired t test between average accuracy of color compared to motion was not significant (paired t-test: $t_{(34)} = 0.96$, $p = .345$, N=35). Error bars represent corrected within subject SEMs [46, 47]. **i-l.** Depicted are some minor interactions of no interest with Context, according to the exploratory model, N=35 for all panels. Error bars and bands in panels g-l correspond to 89% confidence interval. Source data are provided as a Source Data file.

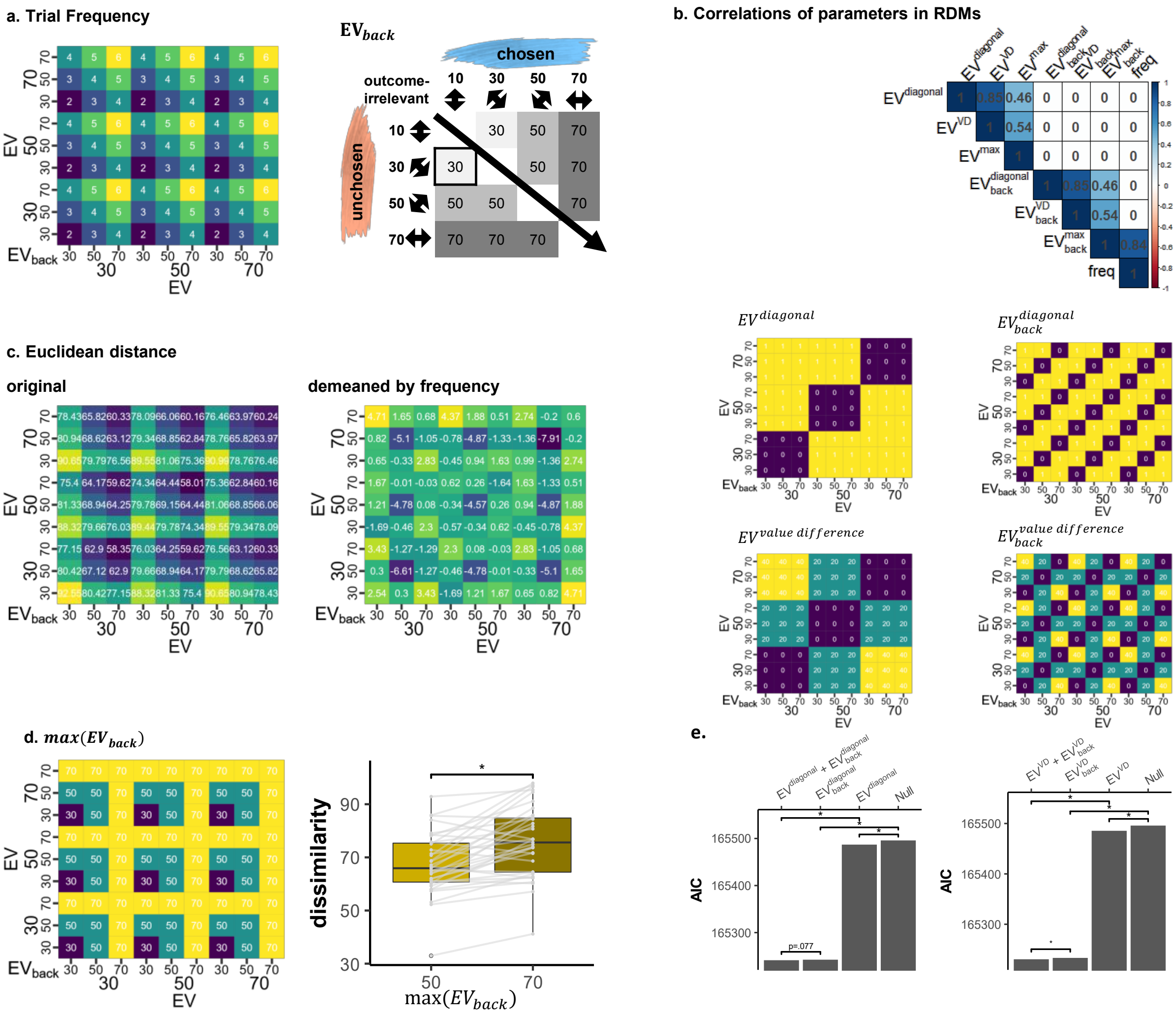

Figure S6: Frequency bias in the design and supplementary information for Representational Similarity Analysis: related to Fig. 5 and Fig. 3

Fig. S6: Frequency bias in the design and supplementary information for Representational Similarity Analysis: related to Fig. 3 **a-b** Panel a shows the frequency of unique examples within 2D trials (for each context). Panel b is taken from Fig. 1e. to help with visualization. Each cell shows the number of how many trials were used for to both the betas that correspond to that cell (presented as ratio relative to the rest). As can be seen, our design included more trials for higher EV_{back}. We believe this is the reason why the probabilities the classifier trained on 2D trials were biased. Note that the analyses depicted in Fig. 5g-i. was conducted nested within the levels of EV_{back}, thus eliminating influences of frequency of trials (henceforth: Frequency) from the probability of the EV_{back} classifier. Additionally, all RSA models were conducted nested within the levels of Frequency, meaning all effects found go beyond any mean difference resulting from the frequency bias. **c** Correlations of parameters used in the RSA analyses show that all the main and value difference parameters are orthogonal to the frequency effect. Added below the correlations are the effects taken from Fig. 3 to help with visualization.

d. In order to replicate the effect found in Fig. 5b, when focusing only on the cells corresponding to the same EV (i.e. corresponding to the diagonal in the EV main effect matrix), only one level of Frequency (4) has two separate levels of $\max_{back}^{EV}$ (parameter indicating which is the maximum EV_{back} involved in the comparison, explaining the high correlation in panel c). Nevertheless, when comparing these two cells across subjects we find a positive effect of $\max_{back}^{EV}$ indicating an increase in dissimilarity of EV representation when $\max_{back}^{EV}$ is higher, paired t-test: $t_{(34)} = -5.42$, $p < .001$, N=35. Boxes mid-line represent median, lower and upper the 25th and 75th percentile and whiskers extend to the range of the data (no more than 1.5 of the full box range). Data beyond the whiskers are plotted individually as solid points. **e.** Hierarchical model comparison showing that the model with both Main effects (right) and with both Value similarity effects (left) explain the data best. All models are nested within the levels of frequency (see panel a). Likelihood-ratio-tests with added terms: For Diagonal effects models (left): adding EV^{diagonal} to null model: $\chi^2_{(1)} = 10.89$, $p = .001$; adding $EV^{\mathrm{diagonal}}_{\mathrm{back}}$ to null model: $\chi^2_{(1)} = 255.44$, $p < .001$; adding EV^{diagonal} to the model with EV^{diagonal}: $\chi^2_{(1)} = 3.12$, $p = .077$. adding $EV^{\mathrm{diagonal}}_{\mathrm{back}}$ to the model with $EV^{\mathrm{diagonal}}_{\mathrm{back}}$: $\chi^2_{(1)} = 247.67$, $p < .001$; For Value Difference models (VD, right): adding EV^{VD} to null model: $\chi^2_{(1)} = 12.34$, $p < .001$; adding $EV^{\mathrm{VD}}_{\mathrm{back}}$ to null model: $\chi^2_{(1)} = 264.61$, $p < .001$; adding EV^{VD} to the model with $EV^{\mathrm{VD}}_{\mathrm{back}}$: $\chi^2_{(1)} = 4.71$, $p = .03$; adding $EV^{\mathrm{VD}}_{\mathrm{back}}$ to the model with EV^{VD}: $\chi^2_{(1)} = 256.98$, $p < .001$. Stars in panels d-e represent the p-value is lower than conventional .05 threshold. Source data are provided as a Source Data file.

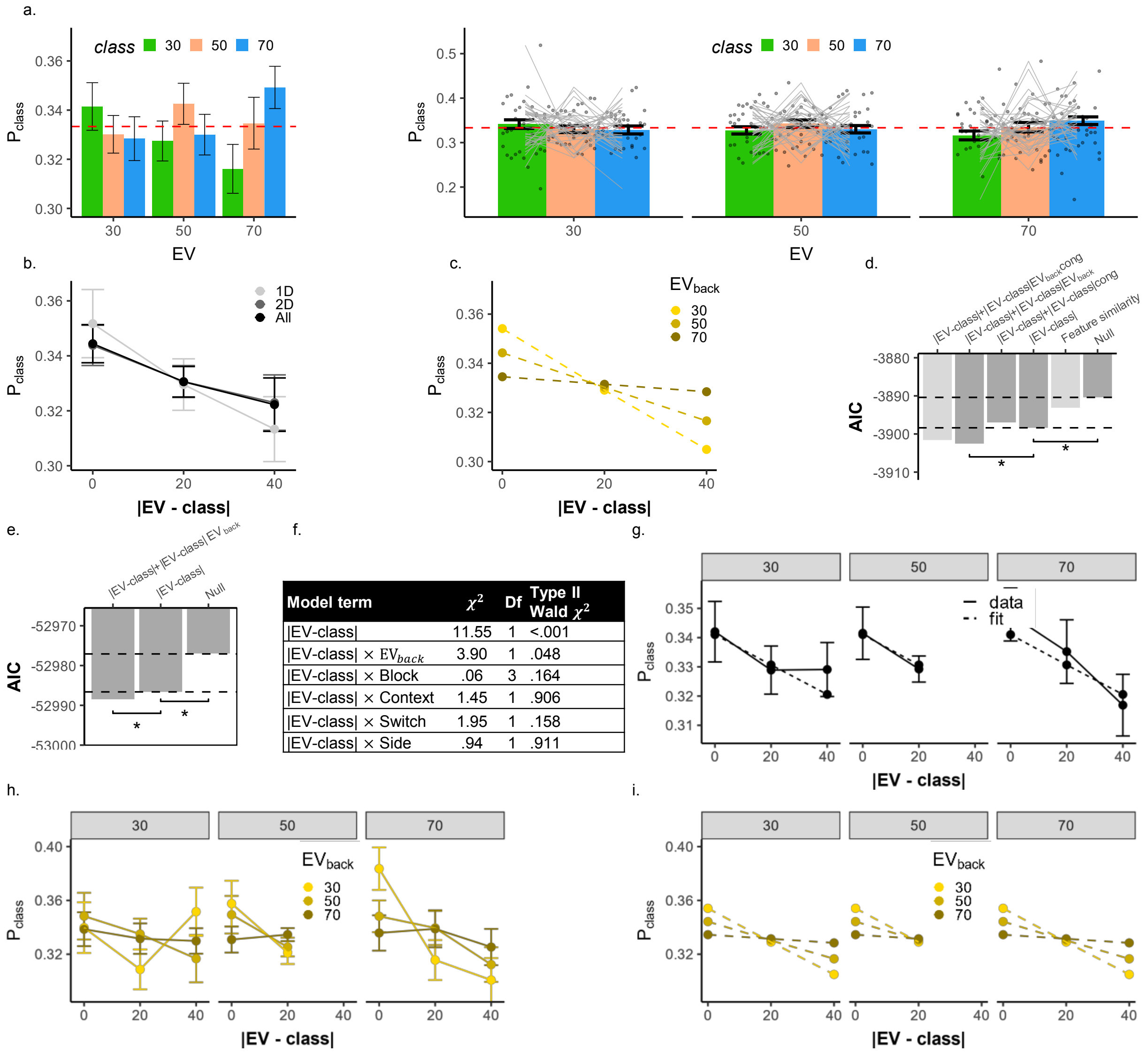

Model term	χ^2	Df	Type II Wald χ^2
|EV-class|	11.55	1	<.001
|EV-class| × EV_{back}	3.90	1	.048
|EV-class| × Block	.06	3	.164
|EV-class| × Context	1.45	1	.906
|EV-class| × Switch	1.95	1	.158
|EV-class| × Side	.94	1	.911

Figure S7: Supplementary information for value similarity analysis: related to Fig. 4 and Fig. 5

Fig. S7: Supplementary information for Value similarity analysis: related to Fig. 4 and Fig. 5

.

Focusing on the Value classifier we asked whether EVs affected not only the probability of the corresponding class, but also influenced the full probability distribution predicted by the Value classifier. We reasoned that if the classifier is decoding the neural code of values, then similarity between the values assigned to the classes will yield similarity in probabilities associated to those classes. Specifically, we expected not only that the probability associated with the correct class be highest (e.g. '70'), but also that the probability associated with the closest class (e.g. '50') would be higher than the probability with the least similar class (e.g. '30', panel a, note that this difference also reflects which options where displayed vs not in a given trial). The following analyses model directly the class probabilities estimated by this classifier.

Probabilities were modelled with beta regression mixed effects models [49]. For technical reasons, we averaged across nuisance regressors used in behavioral analyses. An exploratory analysis of raw data including nuisance variables showed that they had no influence and confirmed all model comparison results reported (see Fig. S7. To test our hypothesis, we modelled the probabilities in each trial as a function of the absolute difference between the objective EV of the trial and the class (|EV-class|, i.e. in the above example with a correct class of 70, the probability for the class 50 will be modelled as condition 70-50=20 and the probability of 30 as 70-30=40). This analysis indeed revealed such a value similarity effect ($\chi^2_{(1)} = 12.74$, $p < .001$) also when tested separately on 1D and 2D trials ($\chi^2_{(1)} = 14.22$, $p < .001$, $\chi^2_{(1)} = 9.99$, $p = .002$, respectively, panel d.). Note that the difference between |EV-class| = 20 and |EV-class| = 40 also reflects which options where displayed vs. not in a given trial. Careful analysis of perceptual overlap, however, indicated that this could not explain our results (see below and SI).

Our main hypothesis was that context-irrelevant values might directly influence neural codes of expected value in the vmPFC. The experimentally manipulated background values in our task should therefore interact with the EV probabilities decoded from vmPFC. We thus asked whether the above described value similarity effect was influenced by EV_{back} and/ or Congruency in 2D trials. Analogous to our RT analyses, we used a hierarchical model comparison approach and tested if the interaction of value similarity with these factors improved model fit. We found that EV_{back}, but not Congruency, modulated the value similarity effect ($\chi^2_{(1)} = 6.16$, $p = .013$, $\chi^2_{(1)} = .58$, $p = .446$, respectively, panel d). This effect indicated that the higher the EV_{back} was, the less steep was the value similarity effect. These results also hold when running the models nested within the levels of EV (panels g-i). Additional control analyses included perceptual models that merely encoded the amount of perceptual overlap between each training class and 2D testing as well as the presence of the perceptual feature corresponding to EV_{back} in the training class. These analyses indicated that our classifier was indeed sensitive to values and not only to the perceptual features the values were associated with, see S8 for details.

a. Analyses of all probabilities by the Value classifier revealed gradual value similarities. The y-axis represents the probability assigned to each class, colors indicate the classifier class and the x-axis represents the trial type (the objective EV of the trial). As can be seen, the highest probability was assigned to the class corresponding to the objective EV of the trial (i.e. when the color label matched the X axis label). N=35. **b.** Larger difference between the decoded class and the objective EV of the trial (x axis) was related to a lower probability assigned to that class (y axis) when tested in 1D, 2D or all trials (likelihood ratio test compared to null model: all $p < .002$, N=35, grey shades). Hence, the multivariate classifier reflected gradual value similarities. Note that when |EV - class|=0, P_{class} is the probability assigned to the objective EV of the trial. **c.** EV_{back} modulated the value similarity effect (likelihood-ratio test with added term: $p = .013$, N=35) indicating weaker simialrity between EV representations for higher EV_{back}. **d.** AIC values of competing models of value probabilities classified from vmPFC. Hierarchical model comparison of 2D trials revealed not only the differences between decoded class and objective EV (|EV-class|) improved model fit (likelihood-ratio test: $p < .002$, N=35), but rather that EV_{back}

modulated this effect. Crucially, Congruency did not directly modulate the value similarity (likelihood-ratio test: $p = .446$, N=35). Asterisks represent p-value lower than conventional .05 threshold. Light gray bars represent models outside the hierarchical comparison. Including a 3-way interaction (with both EV_{back} and Congruency) did not provide better AIC score (-3902.5,-3901.6, respectively). A perceptual model encoding the feature similarity between each testing trial and the training classes (irrespective of values) did not provide a better AIC score than the value similarity model (|EV-class|), see Fig S8 for details. **e.** Main value similarity model comparison replicated when fitting the models to unaveraged data. Adding a term for |EV-class| improved model fit (likelihood-ratio test with added term: $\chi^2_{(1)} = 11.56$, $p < .001$). Adding an additional term for |EV-class| $\times$ EV_{back} further improved the fit (likelihood-ratio test: $\chi^2_{(1)} = 3.86$, $p = .049$, N=35), as in the model reported in panel c). Asterisks represent p-value lower than conventional .05 threshold. **f.** Effect of Nuisance regressors on unaveraged data (t, Side, Switch and Context). Same as Congruency and EV_{back}, all of the nuisance regressors don't discriminate between the classes, but rather assign the same value to all three probabilities from that trial (which sum to 1). We therefore tested if any of them modulated the value similarity effect. As can be seen in the table, none of the nuisance regressors modulated the value similarity effect. **g-i.** Replication of the value similarity model comparison reported in the main text, averaged across nuisance regressors and nested within the levels of EV, i.e. including EV-specific intercepts nested within each within each subject level ($\zeta_{0_{k_v}}$, see methods). As in the analysis reported in the Main Text, adding a main effect for |EV-Class| improves model fit (likelihood-ratio test against null model: $\chi^2_{(1)} = 16.15$, $p < .001$, N=35, first row) as well as adding an additional interaction term |EV-class| $\times$ EV_{back} (likelihood-ratio test with added term: $\chi^2_{(1)} = 6.16$, $p = .013$, N=35). Panel g shows the value simialrity effect across levels of EV, panel h and g show data and fit of the effect of EV_{back} interaction across levels of EV, respectively. Error bars throughout the figure represent corrected within subject SEMs [46, 47]. Source data are provided as a Source Data file.

a.

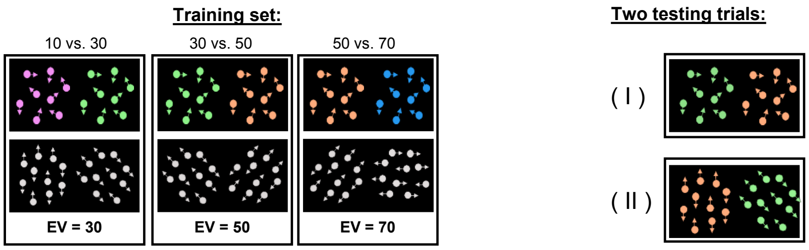

b.

	Test (color)	Test (motion)	*EV*	*EV*$_{back}$	Train Class (color & motion)	|EV-class|	Feature Similarity	*Similarity*$_{back}$
(I) 1D color	30 50		50	--	10 30	**20**	**1** 1x30	-
					30 50	**0**	**2** 1x30, 1x50	-
					50 70	**20**	**1** 1x50	-
(II) 2D color	30 50	10 30	50 color	30 motion	10 30	**20**	**3** 2x30, 1x10	**1**
					30 50	**0**	**3** 2x30, 1x50	**1**
					50 70	**20**	**1** 1x50	**0**

Figure S8: Supplementary information for perceptual similarity analysis: related to Fig. 4 and Fig. 5

Fig. S8: Supplementary information for perceptual similarity analysis: related to Fig. 4. To control that our EV classifier was indeed sensitive to values and not only to the perceptual features the values were associated with, we compared this value similarity model to a perceptual models that merely encodes the amount of perceptual overlap between each training class and 2D testing (irrespective of their corresponding values) and found that our model explained the data best (see panel d). Replacing the $\mathrm{EV_{back}}$ with a parameter that encodes the presence of the perceptual feature corresponding to $\mathrm{EV_{back}}$ in the training class ($\mathrm{Similarity_{back}}$: 1 if the feature was preset, 0 otherwise) did not provide a better AIC score (-3897.1) than including the value of $\mathrm{EV_{back}}$ (-3902.5). **a.** Left: training set consisting of 1D trials provided for the classifier for each class (in the experiment the sides were pseudorandomised). Note that each class had the same amount of color and motion 1D trials and that the value difference between the values was always 20. Right: two examples of 2D trials that constituted the classifier test set. **b.** The table illustrates the calculation of feature similarity between classifier test and training in two example trials in one 1D and one 2D trial. Specifically, shown are the corresponding values and features for each trial with the predicted values at each class for the parameters value similarity (|EV-class|), feature similarity and $\mathrm{similarity_{back}}$. Feature similarity encodes the perceptual overlap between the shown test example and the training examples underlying with each value class. The first row shows a case in which the classifier was tested on a 1D green vs. orange color trial (30 vs 50, EV = 50). Considering in this case for instance the predicted probability that EV=30, the table illustrates the training example underlying the EV = 30 cases (10 vs 30, dark gray shading), the |EV-class| (here: 20, because 50-30), and the feature similarity i.e. how many features from the training class appeared in the test example (here: 1). The second row shows a 2D color trial, reflecting the same value based choice between 30 and 50. The value similarity between training and test stays the same as for the 1D trial shown above. However, the feature similarity between test and training changes because of the motion features. If we take class

30 for example (which is 10 vs 30, dark gray shading), the feature 30 appeared twice (color and motion) and the feature 10 appeared once (motion), i.e. feature similarity now takes on the value 3. $\text{Similarity}_{\text{back}}$ was used to test a perceptual-based alternative to the EV_{back} parameter. $\text{Similarity}_{\text{back}}$ takes on 1 if the perceptual feature corresponding to the EV_{back} appeared in the training class and 0 otherwise (red text in table). As described in the main text, none of the perceptual-similarity encoding alternatives provided a better fit than the reported models that focused on the values the features represent. Source data are provided as a Source Data file.

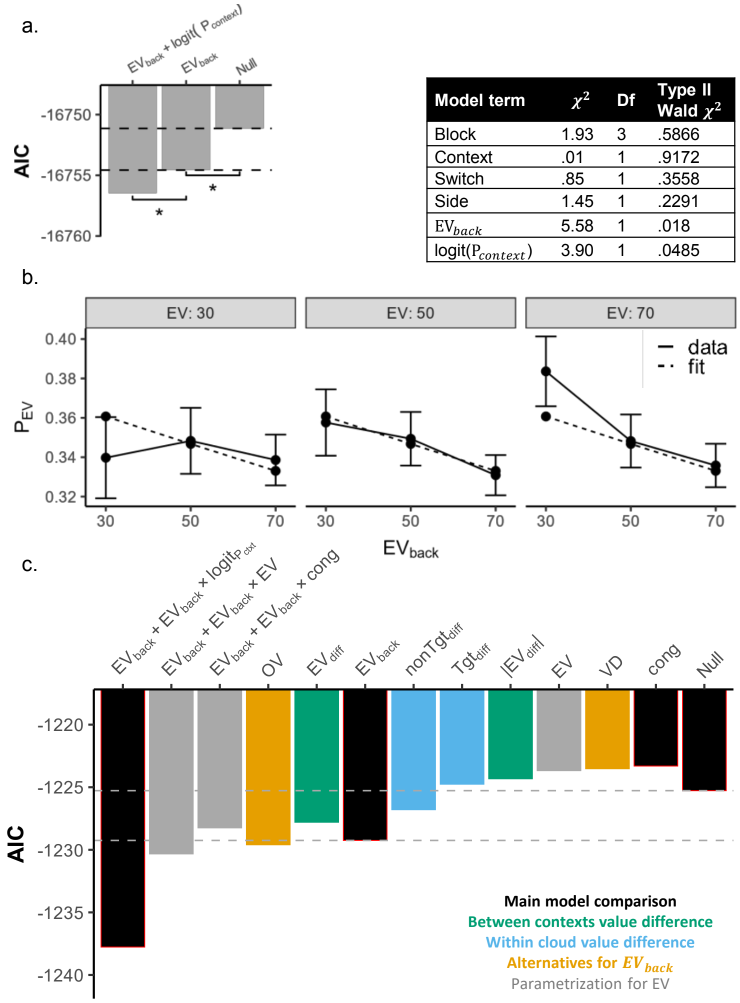

Model term	χ^2	Df	Type II Wald χ^2
Block	1.93	3	.5866
Context	.01	1	.9172
Switch	.85	1	.3558
Side	1.45	1	.2291
EV_{back}	5.58	1	.018
logit($P_{context}$)	3.90	1	.0485

Figure S9: Modelling probability assigned to the EV class.

Fig. S9: Modelling probability assigned to the EV class: related to Fig. 5.
a. We replicated the main results using the unaveraged data. The Null model was: $P^k_{t,EV} = \beta_0 + \gamma_{0k} + \nu_1 side(t) + \nu_2 switch(t) + \nu_3 context(t)$, where $P^k_{t,EV}$ is the probability assigned to the class corresponding to the EV of trial t for subject k, β_0 and γ_{0k} represent global and subject-specific intercepts. Side, Switch and Context are the same as in the RT model (Eq. 2); None of these variables had a main effect, $p > 0.4$ (Type II Wald χ^2 tests, N=35, see table, right), N=35. The factor *trial* could not be included due to model convergence issues. Adding a term representing EV_{back} improved model fit (likelihood-ratio test including term: $\chi^2_{(1)} = 5.42$, $p = .019$). Adding an additional term for context decodability further improved the fit (likelihood-ratio test with added term: $\chi^2_{(1)} = 3.9$, $p = .048$). The table (right) displays the Type 2 Wald χ^2 test for all main effects from the model. **b.** Depicted is the effect of EV_{back} (x-axis) on the probability assignd to the EV class (P_{EV}, y axis). Solid lines represent the data and dashed lines the model fit of a model that included random effects of subject and EV nested within subject (data averaged across nuisance regressors, adding a main effect for EV_{back} improved model fit (likelihood-ratio test with added term: $\chi^2_{(1)} = 5.99$, $p = .014$, N=35). Error bars represent corrected within subject SEMs

[46, 47]. **c.** Similar to our analysis of alternative models of RT, we clustered models reflecting alternative explanations into three conceptual groups (see color legend; cf. Fig. S3a). All models were fitted to the probability assigned to the objective EV in accurate 2D trials, similar to Eq. 7. Each column represents the AIC (y-axis) of a different model (x-axis) where the labels on the x-axis depict all the main effects included in that specific model (i.e. added to the Null, i.e. Eq. 7 without any main effects). We found no evidence that any other parameters explain the data better than the ones we used in the main text. Specifically, only including main effect of EV_{back}, Overall Value of the irrelevant values (OV) and the difference of both EVs (EV_{diff}) provided a better AIC score than the Null model. Note that adding OV (-1229.6) only slightly surpassed EV_{back} (-1229.26). Crucially, the correlation of EV_{back} and OV is very high (Pearson correlation: $\rho = .87$, see main text). We then looked at possible interactions with the EV_{back} effect. Congruency did not seem to modulate the main effect of EV_{back} and adding an interaction term EV $\times$ EV_{back} provided a slightly better AIC (-1230.33), yet this effect was not significant (likelihood-ratio test: $\chi^2_{(1)} = 3.08$, $p = .079$). Section (b) also visualizes this effect. Lastly, adding a term for the Context decodability provided the lowest (i.e. best) AIC score. This exploratory analysis revealed that our model provides the best fit for P_{EV} in all cases except when EV_{back} was replaced with the sum of irrelevant values (-1229.6, -1229.2, respectively, Fig. S9). In contrast, AIC scores of behavioral models' favored EV_{back} as modulator of Congruency, over the sum of irrelevant values (-6626.6, -6619.9, respectively, Fig.S3). However, both parameters were strongly correlated ($\rho = .87$, $\sigma = .004$) and therefore our task was not designed to distinguish between these two alternatives. Source data are provided as a Source Data file.

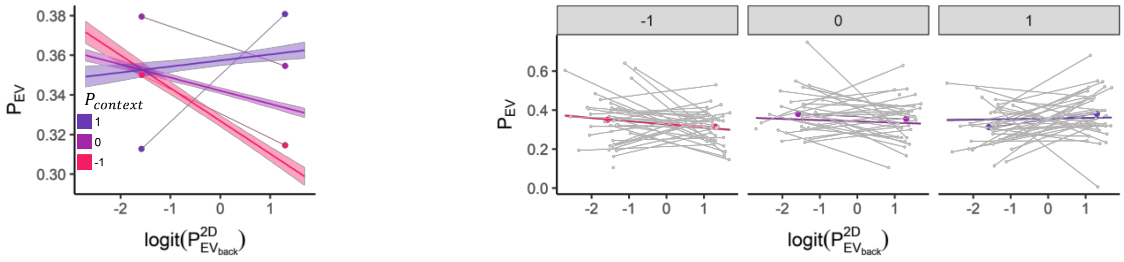

Figure S10: Main effects and corresponding data, fMRI effects, related to Fig. 5

Fig. S10: Main effects and corresponding data, fMRI effects, related to Fig. 5 Since the effects describe data and predictors that are beta-distributed, visualization of simply imposing the true data over the predictions is not very informative. To solve this, and only for visualization purposes here and in the main paper, we took for each effect the mean of top and bottom 20% of the true probabilities from the classifiers (not transformed) for each participant. Context signal ($P_{context}$) moderated the negative effect of EV_{back} decodability ($P^{2D}_{EV_{back}}$) on EV decodability (P_{EV}). Model prediction of multilogit($P^{2D}_{EV_{back}}$) x $P_{context}$ (left, taken from Fig. 5h.) and top and bottom 20% for each subject for three levels of $P_{context}$ (right, the split to three levels is for visualization whereas in the model the predictor was continuous). In all panels error bands represent the 89% confidence interval. Source data are provided as a Source Data file.

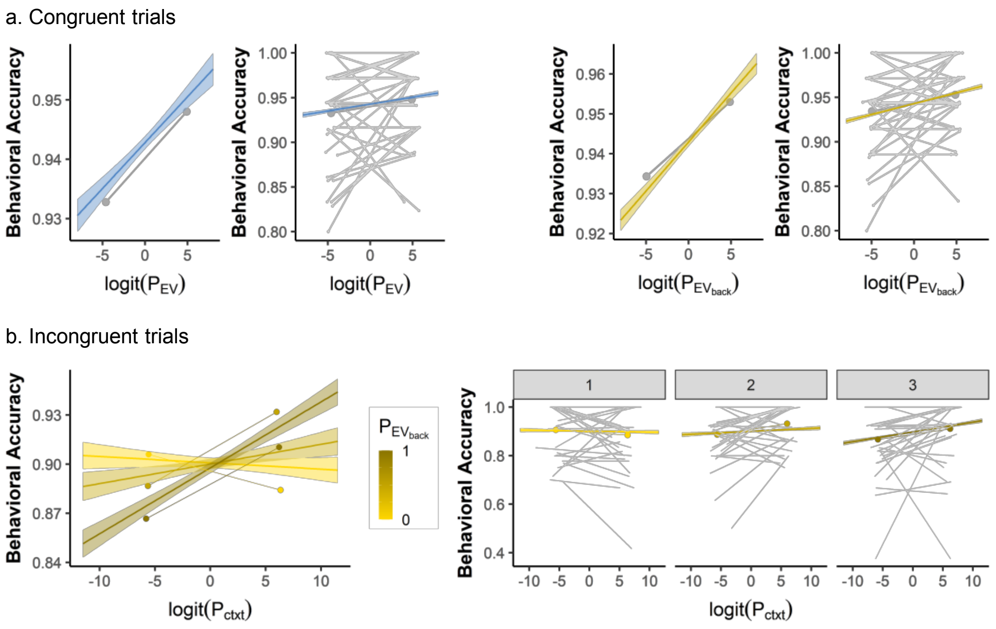

Figure S11: Main effects and corresponding data, link of fMRI to behavioral accuracy, related to Fig. 6

Fig. S11: Main effects and corresponding data, link of fMRI to behavioral accuracy, related to Fig. 6 Since the effects describe data and predictors that are beta-distributed, visualization of simply imposing the true data over the predictions is not very informative. To solve this, and only for visualization purposes here and in the main paper, we took for each effect the mean of top and bottom 20% of the true probabilities from the classifiers (not transformed) for each participant. **a** Congruent trials. Stronger EV decodability (left) and stronger EV_{back} decodability (right) increases behavioral accuracy. The left side of each panel is taken from Fig. 6. The right side depicts the same plot with additional individual subject-specfic lines that represent the top and bottom 20% of the data for each subject (meaning that the gray line on the left side is the mean of the individual lines on the right). **b.** Incongruent trials. Stronger Context decodability ($P_{context}$) increases behavioral accuracy, modulated by EV_{back} decodability ($P_{EV_{back}}$) such that when $P_{EV_{back}}$ was low, the effect of $P_{context}$ diminished. For visualization purpose, Right panel is split by 3 equal sized bins of $P_{EV_{back}}$ (left is the lowest bin, increasing to the right, the split to three levels is for visualization whereas in the model the predictor was continuous). In all panels error bands represent the 89% confidence interval. Source data are provided as a Source Data file.

a. b. c. d.

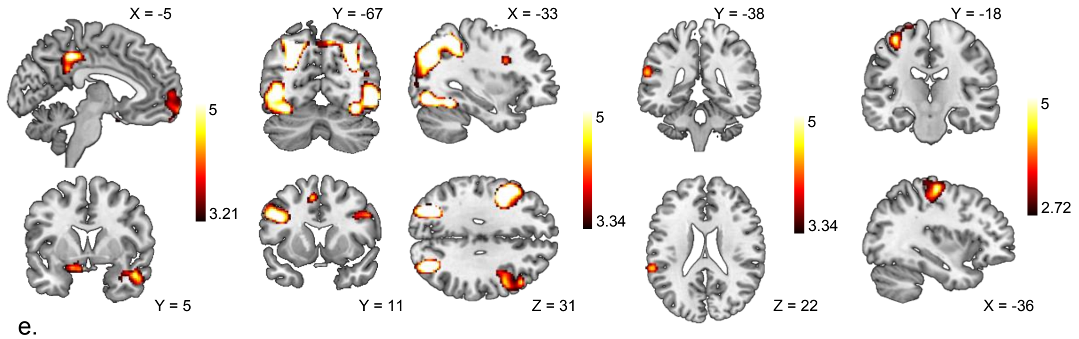

e.

Cue (split)	Accurate Stimuli		Non-accurate stimuli	Outcome	+ 13 fmriprep	+ 18 Physiological
Color + Motion	+ 1D	+ 2D	+ Wrong + no-answer	+ Correct + wrong + no-answer	See online methods	

GLM	Parametric modulators 1D	2D
GLM1	EV	EV
GLM2	EV	EV +EV_{back} +Congruency
GLM3	EV	EV +(EV_{back} x Congruency)
GLM4	EV	(EV x Congruency)

Parametric modulators (demeaned):	
EV	{30, 50, 70}
Congruency	{+1, -1}
EV_{back}	{30, 50, 70}
EV_{back} x Congruency	{-70,-50,-30,30, 50, 70}
EV x Congruency	{-70,-50,-30,30, 50, 70}

GLM	Contrasts: below threshold, *p<0.005, **p<0.001
GLM1	2D > 1D** , 1D > 2D*
GLM2	Congruency > 0, Congruency < 0, EV_{back} > 0, EV_{back} < 0**
GLM3	(EV_{back} x Congruency) > 0, (EV_{back} x Congruency) < 0 *
GLM4	(EV x Congruency) > 0, (EV x Congruency) < 0

Figure S12: Main univariate results

Fig. S12: Main univariate results.

The main analyses indicated that multiple value expectations are represented in parallel within vmPFC. Here, we asked whether whole-brain univariate analyses could also uncover evidence for processing of multiple value representations. In particular, we asked whether we could find evidence for a single representation that integrates the multiple value expectations into one signal. To this end, we first analyzed the fMRI data using GLMs with separate onsets and EV parametric modulators for 1D and 2D trials (see below for detailed description).

a. The intersection of the EV parametric modulators of 1D and 2D trials ($\mathrm{EV}_{\mathrm{1D}} > 0 \cap \mathrm{EV}_{\mathrm{2D}} > 0$) revealed several regions including right Amygdala, bilateral Hippocampus and Angular Gyrus, the lateral and medial OFC and overlapping vmPFC. Hence, the vmPFC signaled the expected value of the current context in both trial types as expected – even though 2D trials likely required higher attentional demands (see panel b). Voxelwise threshold $p < .001$, FDR cluster-corrected. **b** 2D trials were characterized by increased activation in an attentional network involving occipital, parietal and frontal clusters (2D > 1D, $p < .001$ FDR cluster corrected).

Next, we searched for univariate evidence of processing irrelevant values by modifying the parametric modulators assigned to 2D trials in the above-mentioned GLM. Specifically, in addition to EV_{2D}, we added Congruency (+1 for congruent and -1 for incongruent) and EV_{back} as additional modulators of the activity in 2D trials. This GLM revealed no evidence for a Congruency contrast anywhere in the brain (even at a liberal voxel-wise threshold of $p < .005$). **c.** An unexpected negative effect of EV_{back} was found in the Superior Temporal Gyrus ($p < .001$), i.e. the higher the EV_{back}, the lower the signal in this region. $p < .001$, FDR cluster-corrected. No overlap with (b), see S13. We note that this is similar to previous reports implicating this region in modelling choices of others [106]). Notably, unlike the multivariate analysis, no effect in any frontal region was observed.

Motivated by our behavioral analysis, we then turned to look for the interaction of each relevant or irrelevant value with Congruency. An analysis including only a Congruency $\times$ EV_{2D} parametric modulator revealed no cluster (even at $p < .005$).

d. A cluster in the primary motor cortex was negatively modulated by Congruency $\times$ EV_{back}, i.e. the difference between Incongruent and Congruent trials increased with higher EV_{back}, similar to the RT effect and akin to a response conflict, $p < .005$, FDR cluster-corrected. No overlap with (b), see S13

Lastly, we re-ran all above analyses concerning Congruency and EV_{back} only inside the identified vmPFC ROI. No voxel survived for Congruency, EV_{back} nor the interactions, even at threshold of $p < .005$.

e. Visualization of GLMs. The tables depict the structure of GLMs1-4 which were mainly motivated by the behavioral analysis; onset regressors are shown in the top table, parametric modulators assigned to 1D and 2D onsets (middle-left), the values they were modeled with (demeaned, middle-right) are shown below. The contrasts of interest are shown in the bottom table. The GLMs differed only in their modulations of the 2D trials: GLM1 included only modulators of the objective outcome, GLM2 included one modulator for Congruency and one for EV_{back}, GLM3 included a modulator for the Congruency $\times$ EV_{back} interaction and GLM4 included instead of the EV modulator a modulator of the EV $\times$ Congruency interaction. In the contrast table (bottom) contrasts that only revealed effects at a liberal threshold of $p < .005$ are marked with one star, and contrasts significant at $p < .001$ are marked with two stars. All statistical tests represent one-sided t-test either larger or smaller than 0, see lower table in panel e for details of each contrast.

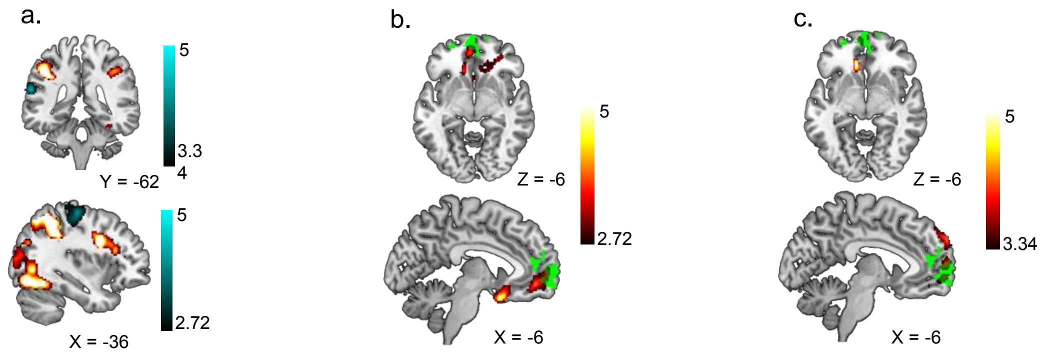

d.

GLM	1D			2D					
GLM5	1D		EV	Congruent		$EV \ +EV_{back}$	Incongruent		$EV \ +EV_{back}$
GLM6	$1D_{30}$	$1D_{50}$	$1D_{70}$	$Cong_{30}$	$Cong_{50}$	$Cong_{70}$	$Incon_{30}$	$Incon_{50}$	$Incon_{70}$

GLM	Contrasts: below threshold, *p<0.005, **p<0.001
GLM5	Congruent > Incongruent, Congruent > Incongruent<br>1D > Incongruent , 1D > Congruent*<br>$Congruent_{EV}$ > $Incongruent_{EV}$,$Congruent_{EV}$ < $Incongruent_{EV}$ *<br>$Congruent_{EV_{back}}$ > $Incongruent_{EV_{back}}$, $Congruent_{EV_{back}}$ < $Incongruent_{EV_{back}}$
GLM6	$Cong_{30}$+$Cong_{50}$+$Cong_{70}$>$Incon_{30}$+$Incon_{50}$+$Incon_{70}$<br>$Cong_{30}$+$Cong_{50}$+$Cong_{70}$<$Incon_{30}$+$Incon_{50}$+$Incon_{70}$<br>$Cong_{70}$>$Incon_{70}$, $Cong_{70}$<$Incon_{70}$*

e.

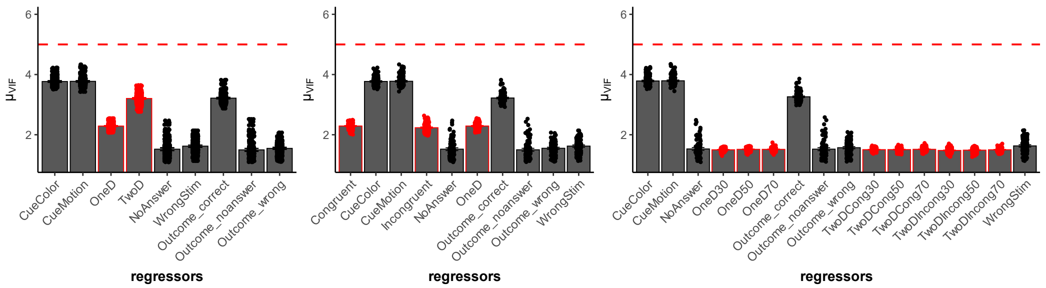

Figure S13: Additional univariate results

Fig. S13: Additional univariate results.
a. Overlap of effects of EV_{back} and trial type (2D > 1D). Main effects of EV_{back}<0 (GLM2, $p < 0.001$ FDR cluster corrected, top, blue shades) and EV_{back} X Congruency < 0 (GLM3, $p < 0.005$, FDR cluster corrected, bottom, blue shades, t values) did not overlap with the 2D network (red shades in both panels, t values). **b.** Main effect of 1D > 2D. A stronger signal in vmPFC for 1D over 2D trials revealed weak activation in a PFC network ($p < .005$, red shades,t values). This included the vmPFC (our functional ROI is depicted in green). Interestingly, at a liberal threshold of $p < .005$ we found stronger activity for 1D over 2D trials in a cluster overlapping with vmPFC (1D > 2D, $p < .005$). Although this could be interpreted as a general preference for 1D trials, splitting the 2D onsets by Congruency revealed no cluster for 1D > Incongruent (also at $p < .005$) but a stronger cluster for 1D > Congruent ($p < .001$,Fig. S13). In other words, the signal in the vmPFC was *weaker* when both contexts indicate the same action, compared to when only one context is present. **c.** Stronger signal in vmPFC for 1D over congruent

but not incongruent trials. When we split the onset of the 2D into Congruent and Incongruent trials (GLM5), we found no significant cluster for the 1D > Incongruent contrast, but an overlapping and stronger cluster for the 1D > Congruent contrast ($p < .001$, FDR cluster corrected, red shades, t values). We found very similar results when contrasting the onsets of 1D and Congruent in GLM6 (not presented), confirming the same results also when controlling for the number of trials for each level of EV (i.e. $1D_{30}$+$1D_{50}$+$1D_{70}$> $Congruent_{30}$+$Congruent_{50}$+$Congruent_{70}$). Our functional ROI is depicted in green. **d.** Additional exploratory analyses such as contrasting the onsets of congruent and incongruent trials, confirmed the lack of Congruency modulation in any frontal region. Specifically, We constructed additional GLMs to verify the results of GLMs 1-4. In GLM5 we split the onset of 2D trials into congruent and incongruent trials and assigned a parametric modulator of EV and EV_{back} to each. As in GLM2, we found no effect of congruency; no voxel survived when contrasting the congruency onsets nor their EV_{back} modulators. Only the contrast $Congruent_{EV}$<$Incongruent_{EV}$ revealed a weak cluster in the right visual cortex (peak 38,-80,16, p<0.005 not presented). In GLM6 we split the onsets of the 1D and 2D trials by levels of EV and the 2D trials further by Congruency. No Congruency main effect survived correction. Only when the onsets of Congruent and Incongruent 2D trials with EV=70 were contrasted, a cluster in the primary motor cortex was found (also at $p < .005$). Unsurprisingly, this cluster largely overlapped with the Congruency $\times$ EV_{back} effect reported in the Main Text. Except the contrast of 1D > Congruent (see Main Text) none of the other contrasts shown in the table revealed any cluster, even at $p < .005$. All statistical tests in panels a-d represent one-sided t-test either larger or smaller than 0, see lower table in panel d for details of each contrast. **e.**Variance Inflation Factor (VIF) of the different regressors in all GLMs. None of the regressors (x axis) had a mean VIF value (y axis) across blocks and participants above the threshold of 4. Regressors involved in GLMs 1-4 shown on the left (Fig. S12); GLM5 and GLM6 are shown in the middle and on the right, respectively. See Methods for details. N=35. Error bars represent corrected within subject SEMs [46, 47]

Anatomical region			Peak (MNI)				peak	
Label		Distance	X	Y	Z	Cluster size	t$_34$	p$_unc$
**$EV_{1D} > 0 \cap EV_{2D} > 0$, p<001, k = 280**								
R	Inferior Temporal Gyrus	4.90	60	-18	-14	1770	6.53	< .0001
R	Middle Temporal Gyrus	0	50	-6	-20		5.49	< .0001
R	Middle Temporal Gyrus	0	56	-30	-8		5.27	< .0001
R	Superior Frontal Gyrus, medial Orbital	0	8	68	-12	1045	6.09	< .0001
L	Inferior Frontal Gyrus pars orbitalis	0	-50	30	-10		4.67	< .0001
L	Superior Frontal Gyrus	0	-24	58	-6		4.35	< .0001
L	Middle Temporal Gyrus	0	-60	-30	-6	1318	5.85	< .0001
L	Middle Temporal Gyrus	0	-66	-24	-8		5.78	< .0001
L	Hippocampus	2	-40	-26	-12		4.96	< .0001
L	Angular Gyrus	0	-50	-60	38	875	5.58	< .0001
L	Angular Gyrus	0	-46	-52	30		4.86	< .0001
L	Angular Gyrus	0	-46	-70	34		3.66	.0002
L	Middle Cingulate & Paracingulate Gyri	0	-4	-40	44	1065	5.51	< .0001
L	Posterior Cingulate Gyrus	0	0	-44	32		4.52	< .0001
R	Middle Cingulate & Paracingulate Gyri	0	12	-48	32		4.52	< .0001
L	Hippocampus	0	-18	-6	-20	280	4.59	< .0001
L	Olfactory Cortex	2	-10	6	-18		4.34	< .0001
R	Angular Gyrus	0	50	-56	30	474	4.27	< .0001
R	Superior Temporal Gyrus	0	62	-54	22		4.26	< .0001
**2D > 1D, p<.001, k=158**								
L	Superior Occipital Gyrus	2.83	-28	-76	38	5367	8.71	< .0001
L	Inferior Occipital Gyrus	0	-48	-76	-4		7.69	< .0001
L	Superior Parietal Gyrus	0	-28	-66	52		7.62	< .0001
L	Precentral Gyrus	0	-46	4	30	1766	7.69	< .0001
L	Inferior Frontal Gyrus, triangular part	0	-44	34	22		5.88	< .0001
L	Inferior Frontal Gyrus, triangular part	0	-40	26	22		5.59	< .0001
R	Inferior Parietal Gyrus	0	32	-56	54	3876	7.23	< .0001
R	Fusiform Gyrus	0	30	-76	-10		7.16	< .0001
R	Inferior Temporal Gyrus	0	48	-70	-8		7.13	< .0001
R	Inferior Frontal Gyrus, triangular part	0	48	26	26	616	5.17	< .0001
R	Precentral Gyrus	0	48	8	32		4.50	< .0001
R	Precentral Gyrus	0	38	2	30		4.23	.0001
L	Supplementary Motor Area	0	-8	14	50	159	4.69	< .0001
**$EV_{back} < 0$, p<.001, k = 240**								
L	SupraMarginal Gyrus	2	-62	-38	22	240	4.50	< .0001
L	Superior Temporal Gyrus	0	-60	-32	10		4.26	.0001
L	Superior Temporal Gyrus	0	-60	-22	8		3.71	.0004
**Congruency $\times$ $EV_{back} < 0$, p<.005, k=632**								
L	Postcentral Gyrus	6.93	-36	-18	60	632	4.03	.0002
L	Postcentral Gyrus	0	-48	-22	52		3.11	.0019
L	Postcentral Gyrus	0	-24	-20	74		3.08	.0020
**$EV_{1D} + EV_{2D} > 0$, within functional ROI, p<.001, k=979**								
R	Anterior Orbital Gyrus	4.47	8	68	-12	979	7.89	< .0001
L	Superior Frontal Gyrus, Medial Orbital	2	-6	68	-12		6.86	< .0001
L	Superior Frontal Gyrus, Medial	0	-10	64	2		5.86	< .0001

Table S1: Detailed univariate results: Clusters for whole brain univariate analysis, related to Fig. S12. Presented are the closest labels to the local maxima of each cluster and each contrast using AAL3v1 [99–101]. All contrasts are FDR cluster corrected. p and k values presented for each cluster. p values represent one sided t-test.

Effect sizes and confidence intervals for best explaining models In all the following tables, CI marks Confidence Interval, CIl and CIh the low and high ends of the confidence interval respectively. P-values in all tables showing best explaining models represent Type II Wald χ^2 tests.

	Parameter	Coef	CI	CIl	CIh	t	df_er	p	Effects	Std_{Coef}	Std^{CIl}_{Coef}	Std^{CIh}_{Coef}
1	(Intercept)	0.08	0.95	0.06	0.11	6.48	9035	0.00	fixed	0.17	0.04	0.29
2	Switch	-0.01	0.95	-0.01	-0.01	-6.57	9035	0.00	fixed	-0.06	-0.08	-0.04
3	Trial	-0.01	0.95	-0.02	-0.01	-6.92	9035	0.00	fixed	-0.06	-0.08	-0.04
4	side [R]	-0.01	0.95	-0.01	-0.00	-2.29	9035	0.02	fixed	-0.04	-0.08	-0.01
5	Context [M]	-0.07	0.95	-0.08	-0.07	-20.73	9035	0.00	fixed	-0.37	-0.40	-0.33
6	EV	-0.07	0.95	-0.08	-0.07	-29.14	9035	0.00	fixed	-0.36	-0.39	-0.34
7	Congruency [1]	0.02	0.95	0.01	0.03	5.40	9035	0.00	fixed	0.10	0.06	0.13
8	Congruency [-1] * EV_{back}	-0.01	0.95	-0.01	-0.00	-2.07	9035	0.04	fixed	-0.03	-0.05	-0.00
9	Congruency [1] * EV_{back}	0.01	0.95	0.00	0.01	3.73	9035	0.00	fixed	0.05	0.02	0.07
10	EV * Congruency [1]	0.01	0.95	0.00	0.01	2.08	9035	0.04	fixed	0.04	0.00	0.07
11		0.07	0.95						random-sub			
12		0.17	0.95						random-Residual			

Table S2: Effect sizes and confidence intervals for best explaining RT model

	Parameter	Coef	CI	CIl	CIh	z	p	Effects	Std_{Coef}	Std^{CIl}_{Coef}	Std^{CIh}_{Coef}
1	(Intercept)	-0.65	0.95	-0.69	-0.61	-30.83	0.00	fixed	-0.65	-0.69	-0.61
2	EV_{back}	-0.05	0.95	-0.08	-0.01	-2.58	0.01	fixed	-0.05	-0.08	-0.01
3	logit($P_{context}$)	0.06	0.95	0.02	0.10	3.25	0.00	fixed	0.06	0.02	0.10
4		0.06	0.95	0.02	0.18			random-sub			
5		8.79	0.95					random-Residual			

Table S3: Effect sizes and confidence intervals for best explaining fMRI model (main model)l

	Parameter	Coef	CI	CIl	CIh	z	p	Effects	Std_{Coef}	Std_{Coef}^{CIl}	Std_{Coef}^{CIh}
1	(Intercept)	-0.65	0.95	-0.69	-0.61	-31.36	0.00	fixed	-0.65	-0.69	-0.61
2	logit($P_{context}$)	0.06	0.95	0.02	0.09	3.02	0.00	fixed	0.06	0.02	0.09
3	mlogit($P_{EV_{back}}$	-0.03	0.95	-0.07	0.01	-1.63	0.10	fixed	-0.03	-0.07	0.01
4	logit($P_{context}$) * mlogit($P_{EV_{back}}$)	0.04	0.95	0.00	0.08	2.17	0.03	fixed	0.04	0.00	0.08
5		0.05	0.95	0.00	0.62			random-EV_{back}:sub			
6		0.04	0.95	0.00	0.70			random-sub			
7		8.82	0.95	8.16	9.54			random-Residual			

Table S4: Effect sizes and confidence intervals for best explaining fMRI model (model nested in EV_{back}

	Parameter	Coef	CI	CIl	CIh	z	df_error	p	Effects	Std_{Coef}	Std_{Coef}^{CIl}	Std_{Coef}^{CIh}
1	(Intercept)	0.01	0.95	0.01	0.02	30.66	Inf	0.00	fixed	0.01	0.01	0.02
2	$EV^{diagonal}$	-0.00	0.95	-0.00	0.00	-1.77	Inf	0.08	fixed	-0.00	-0.00	0.00
3	$EV_{back}^{diagonal}$	-0.00	0.95	-0.00	-0.00	-15.61	Inf	0.00	fixed	-0.00	-0.00	-0.00
4		0.00	0.95	0.00	0.00				random-freq:sub			
5		0.00	0.95	0.00	0.00				random-sub			
6		0.27	0.95	0.27	0.27				random-freq			

Table S5: Effect sizes and confidence intervals for best explaining RSA model - diagonal models

	Parameter	Coef	CI	CIl	CIh	z	df_error	p	Effects	Std_{Coef}	Std_{Coef}^{CIl}	Std_{Coef}^{CIh}
1	(Intercept)	0.01	0.95	0.01	0.02	30.66	Inf	0.00	fixed	0.01	0.01	0.02
2	VDev	-0.00	0.95	-0.00	-0.00	-2.17	Inf	0.03	fixed	-0.00	-0.00	-0.00
3	VDevback	-0.00	0.95	-0.00	-0.00	-15.93	Inf	0.00	fixed	-0.00	-0.00	-0.00
4		0.00	0.95	0.00	0.00				random-freq:sub			
5		0.00	0.95	0.00	0.00				random-sub			
6		0.27	0.95	0.27	0.27				random-freq			

Table S6: Effect sizes and confidence intervals for best explaining RSA model - value difference models
