# Supplementary material for: Task state representations in vmPFC mediate relevant and irrelevant value signals and their behavioral influence
Source: Nat Commun. 2023 May 31;14:3156. doi: 10.1038/s41467-023-38709-w (PMC10232498; doi:10.1038/s41467-023-38709-w)
Supplement: Supplementary file 3 — Reporting Summary [file 41467_2023_38709_MOESM3_ESM.pdf]

nature portfolio

Corresponding author(s): *Double-blind peer review submissions: write DBPR and your manuscript number here instead of author names.*

Last updated by author(s): *YYYY-MM-DD*

Reporting Summary

Nature Portfolio wishes to improve the reproducibility of the work that we publish. This form provides structure for consistency and transparency in reporting. For further information on Nature Portfolio policies, see our Editorial Policies and the Editorial Policy Checklist.

Statistics

For all statistical analyses, confirm that the following items are present in the figure legend, table legend, main text, or Methods section.

n/a	Confirmed	
☐	☒	The exact sample size (*n*) for each experimental group/condition, given as a discrete number and unit of measurement
☐	☒	A statement on whether measurements were taken from distinct samples or whether the same sample was measured repeatedly
☐	☒	The statistical test(s) used AND whether they are one- or two-sided<br>*Only common tests should be described solely by name; describe more complex techniques in the Methods section.*
☐	☒	A description of all covariates tested
☐	☒	A description of any assumptions or corrections, such as tests of normality and adjustment for multiple comparisons
☐	☒	A full description of the statistical parameters including central tendency (e.g. means) or other basic estimates (e.g. regression coefficient) AND variation (e.g. standard deviation) or associated estimates of uncertainty (e.g. confidence intervals)
☐	☒	For null hypothesis testing, the test statistic (e.g. *F*, *t*, *r*) with confidence intervals, effect sizes, degrees of freedom and *P* value noted<br>*Give P values as exact values whenever suitable.*
☒	☐	For Bayesian analysis, information on the choice of priors and Markov chain Monte Carlo settings
☐	☒	For hierarchical and complex designs, identification of the appropriate level for tests and full reporting of outcomes
☐	☒	Estimates of effect sizes (e.g. Cohen's *d*, Pearson's *r*), indicating how they were calculated

Our web collection on statistics for biologists contains articles on many of the points above.

Software and code

Policy information about availability of computer code

Data collection	Behavioral data were collected using a custom task programmed in the commercial software MATLAB [version R2012b/R2017b; Natick, Massachusetts, USA; The MathWorks Inc.] using the open source Psychophysics Toolbox extensions (version 3.0.11; Brainard, 1997, Spatial Vision; Kleiner et al., 2007, Cognitive and Computational Psychophysics; Pelli, 1997, Spatial Vision) and run on a Windows computer. MRI data were acquired using a 32-channel head coil on a 3-Tesla Siemens Magnetom TrioTim MRI scanner (Siemens, Erlangen, Germany). We measured respiration and pulse during each scanning session using pulse oximetry and a pneumatic respiration belt part of the Siemens Physiological Measurement Unit.
Data analysis	Behavioral data was collected using MATLAB (R2017b). MRI data were arranged according to the Brain Imaging Data Structure (BIDS; Gorgolewski et al., 2016) using the HeuDiConv tool (version 0.6.0.dev1; <https://github.com/nipy/heudiconv>). Dicoms were converted to the NIfTI-1 format using dcm2niix (version 1.0.20190410 GCC6.3.0; Li et al., 2016). Facial features were eliminated from structural images using pydeface (version 2.0; <https://github.com/poldracklab/pydeface>). The data quality of all functional and structural MRI acquisitions were evaluated using MRIQC (version 0.15.2rc1; Esteban et al., 2018). Preprocessing of BIDS-converted MRI data was performed using fMRIPrep (version 1.2.2; Esteban et al., 2018, Esteban et al., 2019; RRID:SCR_016216). Several confound regressors were calculated during fMRIprep preprocessing and additional physiological parameters (respiratory and heart rate) were calculated using the Matlab PhysIO Toolbox (open source code available at TAPAS software collection (version 3.2.0): https://www.translationalneuromodeling.org/tapas.). For more details on the fMRIPrep pipeline, please see <https://fmriprep.readthedocs.io/en/1.2.2/workflows.html> and the Methods section of the manuscript.<br>For univariate analyses, BOLD time-series were re-sampled to MNI152NLin2009cAsym standard space in the fMRIPrep pipeline and then smoothed using SPM12 (Friston et al. 2011) with 8mm FWHM (4mm for ROI generation). Transformation of normalized group-level ROI to native space was done using ANTs (version 2.2.0, Avants et al. 2008; RRID:SCR_004757). Multivariate analyses were conducted in native space, and data was smoothed with 4mm FWHM using SPM (Friston 2011). Classification analyses further preprocessing steps of voxel time-series conducted in Nilearn (version 0.6.2, Abraham et al. 2014). See details in Manuscript.

Behavior (3.7)al statistical analysis was conducted using LME models employing the lmer function of the lme4 package (version 1.1.21, Bates et al., 2015) implemented in custom code in R (version 3.6.1, R Core Team, 2019).
First and Second level mass univariate GLMs were conducted in SPM12 (Friston et al. 2011).
Multivariate analysis was conducted using open-source packages from the Python (version 3.7; Python Software Foundation) and statistical analysis was conducted using Generalized Linear Mixed Models using Template Model Builder (glmmTMB for R; version 0.2.0; Magnusson et al. 2017). All softwares libraries and toolboxes are listed in the code reposatory as well as in the manuscript. For completion here is the extensive list (references can be found in manuscript):
R: R-Studio (1.3.959)
Behavioral scripts: ggplot2 3.3.5, gridExtra 3.3.5, lme4 3.3.5, fs 3.3.5, car 3.3.5, plyr 3.3.5, plotly 3.3.5, ggsignif 3.3.5, RColorBrewer 3.3.5, ggthemes 3.3.5, gtable 3.3.5, sjPlot 3.3.5, sjmisc 3.3.5, knitr 3.3.5, kableExtra 3.3.5, MASS 3.3.5, arrayhelpers 3.3.5, dplyr 3.3.5, grid 3.3.5, ggthemes 3.3.5, corrplot 3.3.5, svglite 3.3.5.
fMRI scripts: additionally glmmTMB 3.3.5, ggeffects 3.3.5.
MATLAB:
preprocessing and univariate analyses:
TAPAS 3.2.0, SPM12 (12)
Python: 3.7
preprocessing:
Please see the extensive list and details of fmriprep version: 1.2.6 either in manuscript or on the documentation of fmriprep.
SPM12 (7771),ANTs 2.2.0
decoding:
pandas 1.1.5, numpy 1.19.5, nilearn 0.6.2 (later changed to 0.7.0), sklearn 0.22.0 (later changed to 0.22.2), nipype 1.7.1

For manuscripts utilizing custom algorithms or software that are central to the research but not yet described in published literature, software must be made available to editors and reviewers. We strongly encourage code deposition in a community repository (e.g. GitHub). See the Nature Portfolio guidelines for submitting code & software for further information.

Data

Policy information about availability of data

All manuscripts must include a data availability statement. This statement should provide the following information, where applicable:

- Accession codes, unique identifiers, or web links for publicly available datasets
- A description of any restrictions on data availability
- For clinical datasets or third party data, please ensure that the statement adheres to our policy

Behavioral data can be found in https://git.mpib-berlin.mpg.de/moneta/parallelrepresentation. All individual fMRI datasets can be found at https://gin.g-node.org/nirmoneta/SODIVA and are shared under Creative Commons Attribution-ShareAlike 4.0 International Public License (see LICENSE file in repository). We supply the fMRI data needed to reproduce the findings presented in the manuscript, i.e. conventionally preprocessed data (fmriprep) from the functionally defined vmPFC ROI (smoothed at 4mm and 8mm, in MNI and native space). We additionally share data from various steps of the analyses: defaced T1 images, functionally defined ROIs in MNI and individual native space, preprocessed data ready to be classified including individual classifier decoding results, individual RSAs (see README in https://git.mpib-berlin.mpg.de/moneta/parallelrepresentation for full details on the data folder structure). In case of interest in the whole brain raw data, please contact the corresponding authors. Source data are provided with this paper.

Custom code for the task, behavioral analyses, preprocessing of fMRI data as well as fMRI analyses to reproduce the findings presented in the manuscript have been deposited in https://git.mpib-berlin.mpg.de/moneta/parallelrepresentation under Creative Commons Attribution-ShareAlike 4.0 International Public License (see LICENSE file in repository).

Human research participants

Policy information about studies involving human research participants and Sex and Gender in Research.

Reporting on sex and gender	Gender of participants was self-reported (note that the study was conducted in the German language where there is no clear distinction between sex and gender). We had no reason to suspect any gender differences in the task and therefore did not include this information in the analyses.
Population characteristics	Forty right-handed young adults took part in the experiment (18 women, mean age = 27.6 years, SD= 3.35) in exchange for monetary reimbursement. Beyond common MRI-safety related exclusion criteria (e.g. piercings, pregnancy, large or circular tattoos etc.), we also did not admit participants to the study if they reported any history of neurological disorders, tendency for back pain, color perception deficiencies or if they had a head circumference larger than 58 cm (due to the limited size of the 32-channel head-coil).
Recruitment	Participants were recruited from an internal participant database or through local advertisement. Any potential self-selection bias, if present, cannot be explicitly ruled out since participants freely chose whether they wanted to participate and contact the experimenter based on the public advertisement and announcements sent through the participant database. These biases are, if present, unlikely to affect the results since the experiment was conducted in a within-subjects design (i.e., all participants experienced all conditions).<br>The pseudo-randomized procedure used (see above section on "Randomization") is also unlikely to interact with any self-selection bias, if present.<br>The main effects investigated in this study (fMRI patterns of fast activation sequences) can be considered general and not specific to a population of young and healthy individuals with high education.

Ethics oversight	The study was approved the the ethics board of the Free University Berlin (Ref. Number: 218/2018)

Note that full information on the approval of the study protocol must also be provided in the manuscript.

Field-specific reporting

Please select the one below that is the best fit for your research. If you are not sure, read the appropriate sections before making your selection.

☒ Life sciences ☐ Behavioural & social sciences ☐ Ecological, evolutionary & environmental sciences

For a reference copy of the document with all sections, see nature.com/documents/nr-reporting-summary-flat.pdf

Life sciences study design

All studies must disclose on these points even when the disclosure is negative.

Sample size	No statistical methods were used to predetermine the sample size. Our sample size of 35 was chosen to be larger than common standards. For example, Szucs, & Ioannidis (2020) show that only 3% of experimental fMRI studies had 40 or more participants and only 9.4% had more than 24 participants.<br>Reference: Szucs, D., & Ioannidis, J. P. (2020). Sample size evolution in neuroimaging research: An evaluation of highly-cited studies (1990–2012) and of latest practices (2017–2018) in high-impact journals. NeuroImage, 221, 117164.
Data exclusions	We excluded five participants from the fMRI cohort analysis; one for severe signal drop in the OFC, i.e. more than 15% less voxels in functional data compared to the OFC mask extracted from freesurfer parcellation of the T1 image (see fMRIPrep). One participant was excluded due to excessive motion during fMRI scanning (more than 2mm in any axial direction) and three participants for low performance (see manuscript). In the behavioral-replication two were excluded for the same accuracy threshold.<br>Due to technical reasons, 3 trials (4 in the replication sample) out of the all trials of all participants were excluded since answers were recorded before stimulus was presented and 2 trials (non in the replication) in which RT was faster than 3 SD from the mean (likely premature response).
Replication	Behavioral results were replicated in one attempt of an additional study of 23 participants. The replication was sucessful. All Reaction Time effects were replicated. Behavioral accuracy was mainly replicated with the exception of one small effect. This is likely due to a ceiling effect due to high accuracy (see manuscript, figure S5).
Randomization	Participants were not divided into groups. Stimuli to reward mapping was pseudo-randomized between participants to ensure all within-context mapping combinations appeared at least once (with the exception for clockwise mapping of motion, see manuscript). The design for each participant was pseudo-randomized to control for any motion, order or repetition suppression confound (see manuscript).
Blinding	Blinding is not relevant to our study as we treat data from all participants equally.

Reporting for specific materials, systems and methods

We require information from authors about some types of materials, experimental systems and methods used in many studies. Here, indicate whether each material, system or method listed is relevant to your study. If you are not sure if a list item applies to your research, read the appropriate section before selecting a response.

Materials & experimental systems

n/a	Involved in the study	
☒	☐	Antibodies
☒	☐	Eukaryotic cell lines
☒	☐	Palaeontology and archaeology
☒	☐	Animals and other organisms
☒	☐	Clinical data
☒	☐	Dual use research of concern

Methods

n/a	Involved in the study	
☒	☐	ChIP-seq
☒	☐	Flow cytometry
☐	☒	MRI-based neuroimaging

Magnetic resonance imaging

Experimental design

Design type	Participants performed a random dot-motion paradigm in two phases, separated by a short break (minimum 15 minutes). The first phase was behavioral (ca. 1.5 hours) and the second phase took place in the MRI scanner in an event-related design (ca. 1.5 hours in total, 60 min on task).
Design specifications	The main task consisted of four blocks (109 trials each). Each run was about 15 minutes in length, including a 20 seconds

break in the middle of the block (while the scanner is running) to allow participants a short break. The structure of each trial was as follows: Cue (0.6s) – Fixation (mean: 0.6s, range: 0.5s-2.5s) – Stimuli (1.6s) – Fixation (mean: 3.4s, range: 1.5s-9s) – Outcome (0,8s) – Fixation (mean: 1.25s, range: 0.7s-6s). The durations of the fixation circles were drawn from a truncated exponential distribution. During the structural scan participants performed a task identical in timing structure to the main task except for fixed fixation timings (0.5s ,0.4s and 0.5s respectively).

Timing of trials and ITIs were optimized using variance inflation factor calculations (VIF) of trial-wise regression models. In short, to verify that the individual trials are estimatable and as a control over multi-collinearity (e.g. Mumford et al. 2015), we convolved a design matrix with the HRF for each subject with one regressor per stimuli, two for cues and three for outcomes. We then computed the VIF for each stimulus regressor (i.e. how predictive is each regressor by the other ones). None of the VIFs surpassed 1.57 across all trials and subjects (mean = 1.42, SD=.033). When repeating this analysis with a GLM in which also outcomes were split into trialwise regressors, we found no stimuli VIF larger than 3.09 (mean = 2.64, SD =.132). Note that 1 is the minimum (best) value and 5 is a relatively conservative threshold for collinearity issues (e.g. Mumford et al. 2015). This means that the BOLD responses of individual trials can be modeled separately and should not have collinearity issues with other stimuli nor with the outcome presentation of each trial (see manuscript for more details).

For other behavioral tasks (titration), see manuscript.

Behavioral performance measures

Behavioral performance was assessed by measuring correct button presses (accuracy, i.e. choosing the most rewarding option) and response times. Mean behavioral accuracy was used as the primary indicator to establish that the subjects were performing the task as expected and reaction times were the main behavioral dependent variable of interest.

Acquisition

Imaging type(s)

Structural and Functional

Field strength

3 Tesla

Sequence & imaging parameters

MRI data was acquired using a 32-channel head coil on a research-dedicated 3-Tesla Siemens Magnetom TrioTim MRI scanner (Siemens, Erlangen, Germany) located at the Max Planck Institute for Human Development in Berlin, Germany. High-resolution T1-weighted (T1w) anatomical Magnetization Prepared Rapid Gradient Echo (MPRAGE) sequences were obtained from each participant to allow registration and brain surface reconstruction (sequence specification: 256 slices; TR = 1900 ms; TE = 2.52 ms; FA = 9 degrees; inversion time (TI) = 900 ms; matrix size = 192 x 256; FOV = 192 x 256 mm; voxel size = 1 x 1 x 1 mm). This was followed with two short acquisitions with six volumes each that were collected using the same sequence parameters as for the functional scans but with varying phase encoding polarities, resulting in pairs of images with distortions going in opposite directions between the two acquisitions (also known as the blip-up / blip-down technique). From these pairs the displacements were estimated and used to correct for geometric distortions due to susceptibility-induced field inhomogeneities as implemented in the the fMRIPrep preprocessing pipeline. In addition, a whole-brain spoiled gradient recalled (GR) field map with dual echo-time images (sequence specification: 36 slices; A-P phase encoding direction; TR = 400 ms; TE1 = 4.92 ms; TE2 = 7.38 ms; FA = 60 degrees; matrix size = 64 x 64; 619 FOV = 192 x 192 mm; voxel size = 3 x 3 x 3.75 mm) was obtained as a potential alternative to the method described above. However, this GR frield map was not used in the preprocessing pipeline. Lastly, four functional runs using a multi-band sequence (sequence specification: 64 slices in interleaved ascending order; anterior-to-posterior (A-P) phase encoding direction; TR = 1250 ms; echo time (TE) = 26 ms; voxel size = 2 x 2 x 2 mm; matrix = 96 x 96; field of view (FOV) = 192 x 192 mm; flip angle (FA) = 71 degrees; distance factor = 0, MB acceleration factor = 4). A tilt angle of 30 degrees from AC-PC was used in order to maximize signal from the orbitofrontal cortex (OFC, see Weiskopf et al. 2006). For each functional run, the task began after the acquisition of the first four volumes (i.e., after 5.00 s) to avoid partial saturation effects and allow for scanner equilibrium.

Area of acquisition

Whole-brain images were acquired.

Diffusion MRI ☐ Used ☒ Not used

Preprocessing

Preprocessing software

MRI data were arranged according to the Brain Imaging Data Structure (BIDS), Dicoms converted to the NIfTI-1 format and data quality evaluated using MRIQC. Preprocessing of BIDS-converted MRI data was performed using fMRIPrep (version 1.2.6; Esteban et al., 2018, Esteban et al., 2019, RRID:SCR_016216). fMRIPrep uses a combination of tools from neuroimaging software packages, including FSL, ANTs, Freesurfer and AFNI. Details of the pipeline are reported in the main manuscript based on fMRIPrep's citation boilerplate and can also be found at https://fmriprep.readthedocs.io/en/1.2.6/workflows.html.
For univariate analyses, BOLD time-series were smoothed (after normalization) using SPM12 with 8mm FWHM (4mm for ROI generation).
Multivariate analyses were conducted in native space, and data was smoothed with 4mm FWHM using SPM. Classification analyses further preprocessing steps of voxel time-series conducted in Nilearn: First, extreme-values more than 8 standard deviations from a voxels mean were corrected by moving them by 50% their distance from the mean towards the mean (this was done to not bias the last z scoring step). Second, the time-series of each voxel was detrended, a high-pass filter at 128 Hz was applied and confounds were regressed out in one action using Nilearn. Lastly, the time-series of each voxel for each block was z scored.
For versions and details see Data Analysis section above and in the manuscript.

Normalization

For univariate analyses, BOLD time-series were re-sampled to MNI152NLin2009cAsym standard space in the fMRIPrep pipeline. Specifically, spatial normalization to the ICBM 152 Nonlinear Asymmetrical template version 2009c [RRID:SCR_008796] was performed through nonlinear registration with antsRegistration [ANTs 2.2.0, RRID:SCR_004757],

using brain-extracted versions of both T1w volume and template. Brain tissue segmentation of cerebrospinal fluid (CSF), white-matter (WM) and gray-matter (GM) was performed on the brain-extracted T1w using fast[FSL 5.0.9, RRID:SCR_002823]. Transformation of normalized group-level ROI to native space was done using ANTs.

Normalization template

The ICBM 152 Nonlinear Asymmetrical template version 2009c (RRID:SCR_008796; Fonov et al., 2009, NeuroImage) was used for nonlinear normalization.

Noise and artifact removal

Several confound regressors were calculated during fMRIprep preprocessing: Six head-motion estimates, Framewise displacement, six anatomical component-based noise correction components (aCompCorr). Additional 18 physiological parameters (8 respiratory, 6 heart rate and 4 of their interaction) were calculated using the Matlab PhysIO Toolbox. All confound regressors were included as nuisance regressors in the first level GLMs as well as regressed out in the preprocessing of the multivariate analysis (see Preprocessing above).

Volume censoring

No volume censoring was performed.

Statistical modeling & inference

Model type and settings

We conducted multivariate leave-one-run-out cross-validated pattern classification analysis where we trained a multinomial logistic regression classifier on functionally defined ROI (via mass-univariate analysis). We also conducted mass-univariate analysis (1st and 2nd level) mostly with 2nd level group t test. For details see “Multivariate modeling and predictive analysis” below and the manuscript.

Effect(s) tested

The following effects were tested: Behavioral RT, accuracy and cross-validated classification accuracies compared to chance using one sample t tests. Main effects of models described with chi-square representing Type II Wald chi-square tests, whereas when describing model comparison, the chi-square represents the log-likelihood ratio test. Mass univariate group results uses one sample or paired t test. For the correlation of predicted probability of the two main EV classes, probabilities were first multinomial logit and then Fisher z-transformed and averaged across trials to achieve one correlation value per subject (Spearman rank correlation). For the link of predicted probabilities to behavioral effects we also used Spearman rank correlation.

Specify type of analysis: ☐ Whole brain ☐ ROI-based ☒ Both

Anatomical location(s)

Group ROI was defined functionally in MNI space using a mass-univariate analysis and was then transformed to the individual native space for the multivariate analysis.

Statistic type for inference
(See Eklund et al. 2016)

Average or ROI-based tests

Correction

Corrections for multiple comparisons were performed by controlling the false discovery rate (FDR) and using the Bonferroni correction with p values reported in the manuscript (usually $p < 0.001$).

Models & analysis

n/a	Involved in the study
☒	☐ Functional and/or effective connectivity
☒	☐ Graph analysis
☐	☒ Multivariate modeling or predictive analysis

Multivariate modeling and predictive analysis

Multivariate modeling was done using multivariate multinomial logistic regression as implemented in scikit-learn 0.22.2 (Pedregosa et al. 2011) set to multinomial (in opposed to one-vs-all) with C-parameter 1.0, lbgfs solver with a 'l2' penalty for regularization.
The training set for all analyses consisted of fMRI data from behaviorally accurate 1D trials. For each trial, we took the TR corresponding to approx. 5 seconds after stimulus onset to match the peak of the Haemodynamic Response Function (HRF) estimated by SPM. Classification training was done using a leave-one-run-out scheme across the four runs with 1D trials. To avoid bias in the training set after sub-setting only to behaviorally accurate trials (i.e. over-representation of some information) we up-sampled each training set to ensure equal number of examples in the training set for each combination of EV (3), Context (2) and Chosen-Side (2). Specifically, if one particular category was less frequent than another (e.g., more value-30, left, color trials than value-50, left-color trials) we up-sampled that example category by randomly selecting a trial from the same category to duplicate in the training set, whilst prioritizing block-wise balance (i.e., if one block had 2 trials in the chunk and another block had only 1, we first duplicated the trial from under-represented block etc.). We did not up-sample the testing set.
The classifier provided for each trial in the testing block one probability (or: predicted probability) per class that was given to it. To avoid bias in the modeling of the classifier's predictions (i.e. one probability for each class) we performed outlier-correction, i.e. rounded up values smaller than 0.00001 and down values bigger than 0.99999. See full preprocessing for Multivariate analysis above.
Multivariate analysis was conducted on features extracted from a mass-univariate defined ROI. In order to generate a functional ROI corresponding to the vmPFC in a reasonable size, we ran a GLM with only relevant Expected Value modulators (i.e. this GLM had no information regarding the contextually irrelevant context which corresponded to the main hypothesis of the paper) on data that was smoothed at 4mm. We then threshold the EV contrasts for 1D and 2D trials (EV_1D + EV_2D>0) at p<.0005. The group ROI was generated in MNI space and included 998 voxels. Multivariate analyses were conducted in native space and the ROI was transformed to native space using ANTs and nearest neighbor interpolation [ANTs 2.2.0] while keeping only

voxels within the union of subject- and run-specific brain masks produced by the fMRIprep pipeline. The resulting subject-specific ROIs therefore had varying number of voxels (mu = 768.14, SD = 65.62, min = 667, max = 954).
